# Identifying and predicting gait stability metrics in people with stroke in uneven-surface walking using machine learning

**DOI:** 10.1038/s41598-026-35966-9

**Published:** 2026-01-17

**Authors:** Yasuhiro Inui, Yusaku Takamura, Yuki Nishi, Shu Morioka

**Affiliations:** 1 Department of Neurorehabilitation, Kio University, Nara, Japan; 2 Department of Rehabilitation, Nara Prefecture General Rehabilitation Center, Nara, Japan; 3 Neurorehabilitation Research Center, Kio University, Nara, Japan; 4 Institute of Biomedical Sciences (Health Sciences), Nagasaki University, Nagasaki, Japan

**Keywords:** Stroke, Gait stability, Uneven surface walking, Machine learning, Trunk acceleration, Cerebrovascular disorders, Predictive markers, Rehabilitation

## Abstract

People with stroke (PwS) face increased fall risk on uneven surfaces; however, gait stability under such conditions remains unexplored. This study used machine learning (ML) to identify acceleration features distinguishing PwS from healthy controls (HC) during uneven-surface walking and to predict them from even-surface gait parameters. Trunk acceleration data from 71 PwS and 39 HC were analyzed using classification and regression models. The ML classifiers achieved an accuracy of over 95%. The key discriminative features included the vertical root mean square (RMS_VT), anterior-posterior sample entropy (SampEn_AP), and harmonic ratio (HR_AP). In PwS, even-surface gait speed < 0.8 m/s predicted reduced speed and higher RMS_VT on uneven surfaces. SampEn_AP and HR_AP were influenced by ankle kinematics and their even-surface values, respectively, showing nonlinear associations. These findings support the use of wearable sensor data and interpretable ML to assess gait stability and adaptability, facilitating development of digital biomarkers for personalized stroke rehabilitation aimed at improving outdoor mobility.

**Supplementary Information:**

The online version contains supplementary material available at 10.1038/s41598-026-35966-9.

## Introduction

Outdoor mobility is essential for community participation and quality of life (QOL) in people with stroke (PwS)^1^. However, many PwS report difficulties walking outdoors^2^, and navigating uneven surfaces poses particular challenges, increasing the risk of falls and mobility limitations^3^. Uneven surface presents unpredictable perturbations that place greater demands on the neuromuscular system, making it more challenging to maintain gait stability^4,5^—the capacity to sustain steady walking despite minor disturbances or errors in control^6^. As PwS often exhibit reduced adaptability, particularly when exposed to such perturbations^7^, the assessment of gait stability in uneven-surface walking is of critical clinical relevance. Yet, this aspect remains underexplored.

Recent advances in wearable sensing have allowed for a detailed analysis of trunk acceleration during walking using inertial measurement units (IMUs) attached to the lower back^8^. Gait stability features can be derived from these signals and categorized into linear and non-linear metrics. Linear analysis commonly uses the root mean square (RMS) of acceleration to quantify the magnitude of variability. PwS exhibited greater trunk RMS values on uneven surfaces than healthy controls, indicating reduced stability. However, the RMS captures only the variability magnitude and not temporal structure. Nonlinear metrics address this issue by characterizing signal dynamics and time-dependent patterns. These include the harmonic ratio (HR) for smoothness, short-term Lyapunov exponent (sLE) for local dynamic stability, recurrence quantification analysis (RQA) for periodicity, and sample entropy (SampEn) for the regularity of the signal. These measures complement linear metrics. In unpredictable environments, such as uneven surfaces, assessing both variability and temporal organization is crucial. The integration of linear and nonlinear metrics offers a more comprehensive understanding of gait stability in PwS. To interpret these indicators effectively, their interrelationships must be clarified using an integrative analysis.

Previous studies that assessed gait stability on uneven surfaces using IMU sensors primarily evaluated each acceleration-derived metric independently^4,9,10^. However, analyzing multiple features separately can lead to redundancy, increased processing time, and over-representation of correlated metrics, potentially hindering their clinical interpretation. Uneven surfaces expose individuals to unpredictable perturbations, to which PwS respond heterogeneously. These responses are often coupled nonlinearly, which limits the explanatory power of traditional linear statistics. Machine learning (ML) addresses these challenges by extracting complex patterns from high-dimensional data and identifying informative features¹²^,^¹³. ML can uncover latent relationships missed by conventional methods and has been increasingly used to distinguish individuals with neurological disorders from healthy controls using gait data^14,15^. Furthermore, combining ML-based automated gait classification with IMU-derived data allows for the rapid and clinically meaningful evaluation of gait abnormalities in individuals with motor impairments^16,17^. A comparative analysis of multiple ML models has been proposed to identify the most accurate classifiers^18,19^, with robust results emerging from a consensus across diverse algorithms. Models range from interpretable “glass box” approaches (e.g., logistic regression (LR)) to less transparent “black box” models (e.g., support vector classifier (SVC), random forest (RF)) ²⁰. Additionally, sparse partial least squares discriminant analysis (sPLS-DA), which integrates feature selection, can be useful for improving model interpretability^21^. Clinical datasets often suffer from limited and imbalanced samples, which can degrade model performance²². To address this, data augmentation techniques such as the Synthetic Minority Over-sampling Technique (SMOTE)²⁵, Generative Adversarial Networks (GAN) ²⁶, and Conditional Tabular GAN (ctGAN)²⁷ have shown promise in improving model accuracy and revealing hidden structures ²³,²⁴. Comparing augmentation methods with ML models helps identify optimal combinations²⁸. Based on these strategies, this study combined data augmentation and ML to identify acceleration-based features that characterize decreased stability in PwS during uneven-surface walking. Standardizing environmental conditions for uneven-surface walking is challenging, making such assessments uncommon in clinical settings. In contrast, gait parameters such as speed, trunk acceleration, muscle activity, and joint angles during even-surface walking are increasingly accessible in practice owing to recent technological advances^29^. Predicting uneven-surface gait stability using these parameters may help guide rehabilitation and improve outdoor mobility. ML regression models can estimate values that normally require specialized environments³⁰ and can be used to assess disease severity in neurological conditions^31^. These include linear (e.g., linear regression and support vector regression (SVR)) and nonlinear methods (e.g., RF and XGBoost). Interpretation tools such as partial dependence plots (PDPs) and SHapley Additive explanations (SHAP), including SHAP + PDP, reveal influential predictors and feature interactions^32^. Applying these techniques to even-surface gait data may help identify key contributors to uneven-surface performance, thereby supporting safer and more adaptive outdoor walking.

The primary aim of this study was to analyze trunk acceleration during uneven-surface walking using multiple machine learning models and evaluate how RMS and nonlinear metrics (HR, RQA, SampEn, and sLE) distinguish PwS from HC. The secondary aim was to predict key acceleration features and uneven-surface gait speed from even-surface gait parameters, including speed, trunk acceleration, electromyography (EMG), and joint angles. By integrating multiple stability metrics derived from wearable sensors with explainable machine learning, this study captures gait instability on uneven surfaces from multiple perspectives and enables prediction based on clinically accessible even-surface walking data, rather than merely describing surface-specific characteristics. This approach may contribute to the development of interpretable digital biomarkers that support the formulation of individualized rehabilitation strategies aimed at improving outdoor mobility in PwS.

## Results

### Participant characteristics in the HC and PwS groups

The demographic and clinical characteristics of the patients are presented in Table 1. No significant differences were found between the two groups in age, height, weight, or Body Mass Index (BMI) (Welch’s tests, all *p* > 0.05), or in sex distribution (Fisher’s exact test, *p* > 0.05). The descriptive statistics for all the gait parameters are shown in Supplementary Table S1.

**Table 1:** Participant demographic characteristics and comparison of variables between the healthy controls (HC) and people with stroke (PwS) groups.

Variable	HC (*n* = 39)	PwS (*n* = 71)
Age (years)^a^	65.6 ± 7.4	63.8 ± 14.2
Sex (male/female)	28/11	55/16
Height (cm)^a^	162.7 ± 8.5	164.8 ± 7.5
Weight (kg)^a^	60.4 ± 8.5	64.1 ± 11.2
BMI (kg/m^2^)^a^	22.8 ± 2.2	23.6 ± 3.6
Time since stroke (days)^a^		62.0(52.0)
FMA-LE^b^		32(5.5)
Berg Balance Scale^b^		54 (7.5)
Gait Efficacy Scale^b^		8 (4)
Non-paretic side Knee extenstion HHD^a^		42.6 ± 12.3

BMI, Body Mass Index; FMA-LE, Fugl-Meyer Assessment Lower Extremity; BBS, Berg Balance Scale; HHD, Hand Held Dynamometer.

^a^: Mean ± Standard deviation, ^b^: Median (Inter quartile range).

### Step 1. stroke detection

#### Glass box model and black box model

##### Feature selection results

Among the acceleration-based features during uneven-surface walking, no pairwise correlations exceeded 0.9 (Supplementary Fig. S1). The Boruta algorithm selected nine features (Supplementary Fig. S2a), whereas the LASSO algorithm selected 11 features (Supplementary Fig. S2b). Eight features—RMS in the anterior–posterior (RMS_AP), mediolateral (RMS_ML), and vertical (RMS_VT) directions; HR in the anterior–posterior direction (HR_AP); SampEn in the anterior–posterior direction (SampEn_AP); %determinism in RQA in the mediolateral direction (RQA_det_ML); and sLE in the anterior–posterior direction (sLE_AP)—were selected by both methods and used as inputs for subsequent supervised ML algorithms.

##### Supervised ML classification metrics

**Fig. 1:**
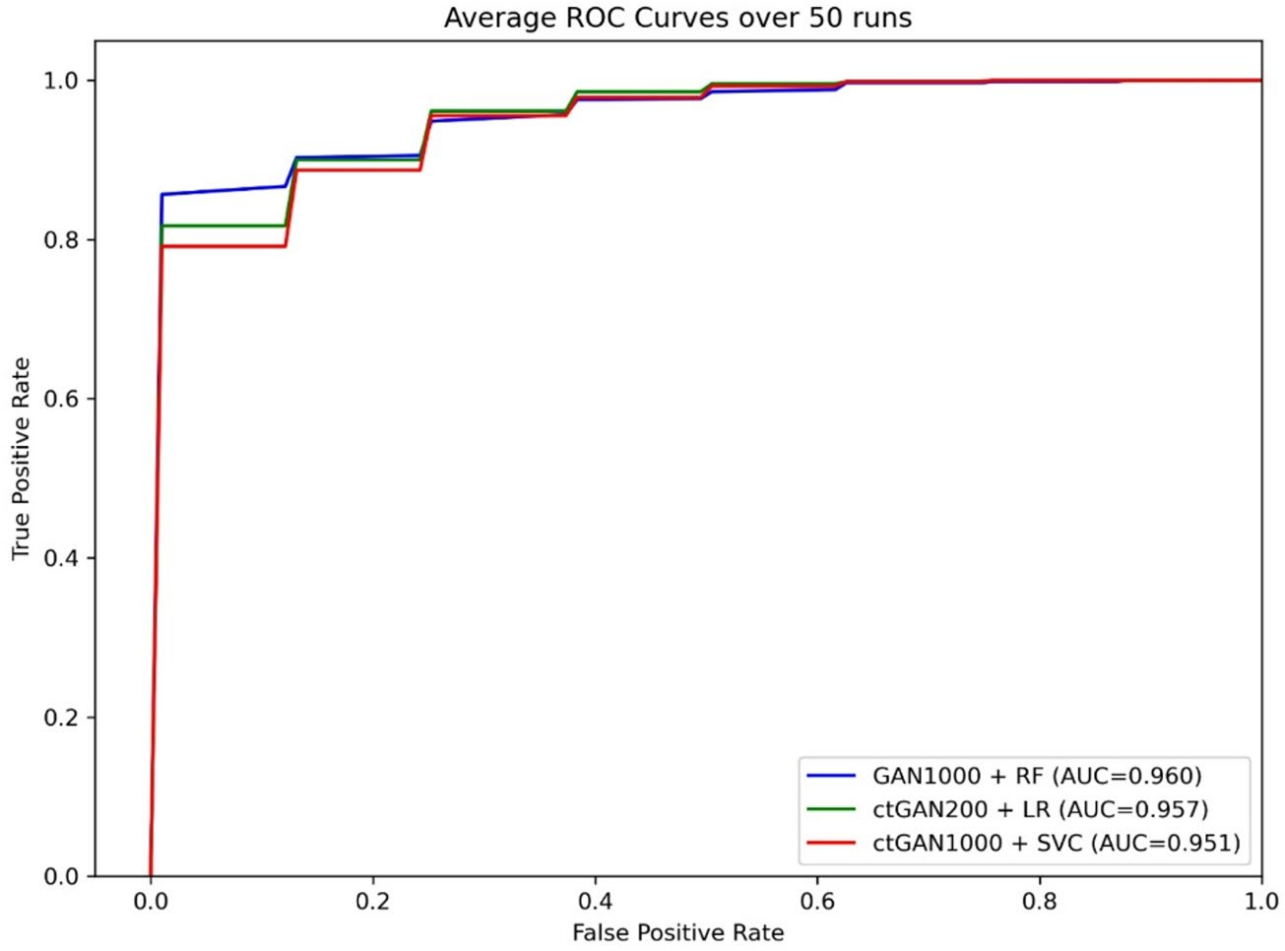
Average ROC curves for top-performing models (50 repeated stratified samplings). RF, Random forest; LR, Logistic Regression; SVC, Support Vector Classification.

Classification performance was evaluated across all combinations of data augmentation strategies and ML models based on the mean values from 50 repeated stratified random samplings. Figure 1 shows the average Receiver Operating Characteristic (ROC) curves for the top-performing combinations: RF with GAN (*N* = 1000), LR with ctGAN (*N* = 200), and SVC with ctGAN (*N* = 1000). A full comparison of the classification metrics for all 36 combinations is presented in Supplementary Table S2.

##### Model interpretation

**Fig. 2:**
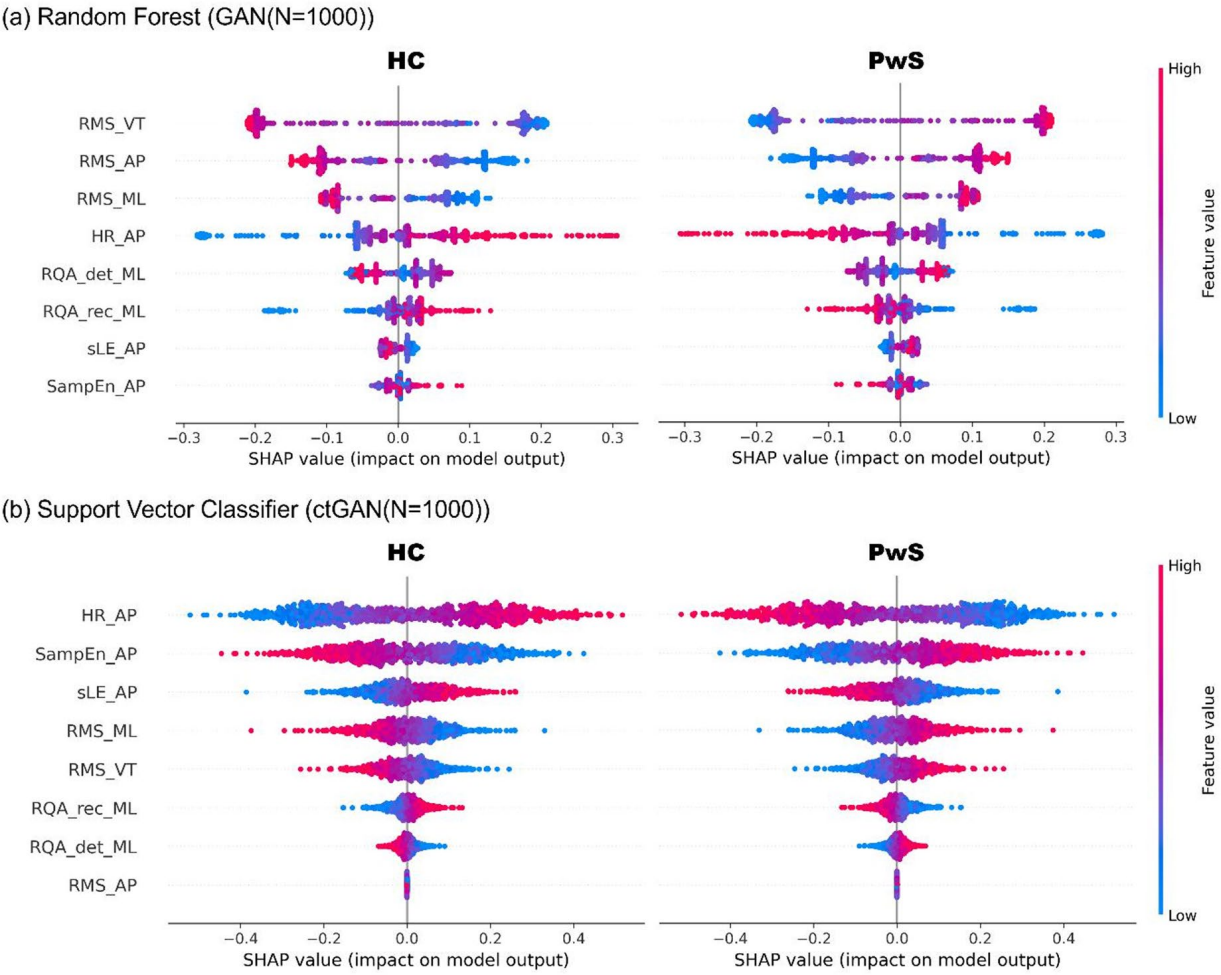
SHAP Value Plot. The x-axis represents the SHAP value of each feature, indicating its contribution to the predicted class. In this visualization, SHAP values are displayed separately for HC (left) and PwS (right), where positive values indicate movement of the model output toward correctly predicting the displayed class (e.g., positive SHAP values on the HC panel increase the likelihood of being classified as HC). The color scale indicates feature magnitude (red = high, blue = low). For example, a high-value feature (red) located on the positive side of the HC plot indicates high feature values increase classification toward HC, whereas a similar pattern in the PwS plot reflects increased likelihood of PwS classification. (**a**) RandomForest (GAN(*N* = 1000)); (**b**) Support Vector Classifier (ctGAN(*N* = 1000)).

Model interpretation was performed for the top-performing models. For LR using ctGAN (*N* = 200), odds ratios were calculated (Table 2). SHAP values were computed for the RF with GAN (*N* = 1000) and SVC with ctGAN (*N* = 1000) (Fig. 2). RMS_VT, a linear acceleration metric, showed the highest importance in both the LR and RF models. Among the nonlinear metrics, SampEn_AP was more important than RMS_ML and RMS_AP in LR, whereas HR_AP was particularly influential in the RF model. In the SVC model, HR_AP demonstrated the highest overall importance, exceeding that of all RMS features.

**Table 2:** Odds ratio in logistic regression (*N* = 200).

Feature	Odds Ratio
RMS_VT	10.30
SampEn_AP	3.55
RMS_AP	2.38
sLE_AP	1.66
RQA_rec_ML	1.52
RMS_ML	1.39
RQA_det_ML	0.49
HR_AP	0.34

RMS, root mean square; SampEn, sample entropy; sLE, short-term maximum Lyapunov exponent; RQA_rec, %recurrence in recurrence quantification analysis; RQA_det, %determinism in recurrence quantification analysis; HR, harmonic ratio; AP, ML, VT, anteriorposterior, mediolateral, and vertical direction of the acceleration signal, respectively.

##### Including features selection model

The sPLS-DA model was used to classify the PwS and HC groups. Component 1, which included RMS_VT, RMS_AP, HR_AP, and RMS_ML, achieved a mean AUC of 0.953 (SD = 0.002). Component 2, consisting of SampEn_AP, RQA_rec_ML, SampEn_ML, and RQA_det_AP, did not contribute to further improvement (mean AUC = 0.953, SD = 0.006), indicating that Component 1 alone was sufficient to achieve high classification accuracy. The distributions of the samples projected onto components 1 and 2 are shown in Supplementary Fig. S3 Component 1, which explained 27% of the variance, contributed the most to group separation, whereas Component 2 (10%) contributed less because of the greater overlap between groups. Although the 95% confidence ellipses partially overlapped, overall separation was evident. Table 3 lists the features used in the model and their corresponding variable importance in projective (VIP) scores. Among the linear metrics, RMS_VT had the highest score, whereas HR_AP, a nonlinear metric, also showed a high score.

**Table 3:** Variable importance in projection (VIP) scores of the sPLS-DA model.

Variable	VIP_Score
RMS_VT	3.004349
RMS_AP	2.693259
HR_AP	1.244307
RMS_ML	1.082562

RMS, root mean square; HR, harmonic ratio; SampEn, sample entropy; RQA_rec, %recurrence in recurrence quantification analysis; RQA_det, %determinism in recurrence quantification analysis; sLE, short-term maximum Lyapunov exponent; AP, ML, VT, anteriorposterior, mediolateral, and vertical direction of the acceleration signal, respectively.

### Step 2. stroke uneven prediction

#### Acceleration indices on uneven surface walking to be predicted

Based on the results of the multiple ML classification models in Step 1, three acceleration indices were identified as the key features distinguishing PwS from HC: RMS_VT, SampEn_AP, and HR_AP. In addition to these indices, uneven-surface gait speed was included as a target variable to be predicted using the regression models in Step 2.

### Feature selection results

No significant correlations were observed among the baseline data. Furthermore, no combination of gait parameters measured during even-surface walking showed correlation coefficients exceeding 0.9 (Supplementary Fig. S4). Supplementary Fig. S5 illustrates the results of feature selection using Boruta and Lasso for each outcome. Specifically, Boruta and Lasso selected 13 and 15 features, respectively, for the even-surface gait speed (S5Aa, S5Ab); 12 and 16 features for RMS_VT (S5Ba, S5Bb); 5 and 11 features for SampEn_AP (S5Ca, S5Cb); and 10 and 6 features for HR_AP (S5Da, S5Db). The optimal regularization parameters used in Lasso are indicated by the corresponding log(λ) values. Features selected by both methods are summarized in Supplementary Table S3 and were used for subsequent supervised machine learning regression.

### Supervised ML regression metrics

**Fig. 3:**
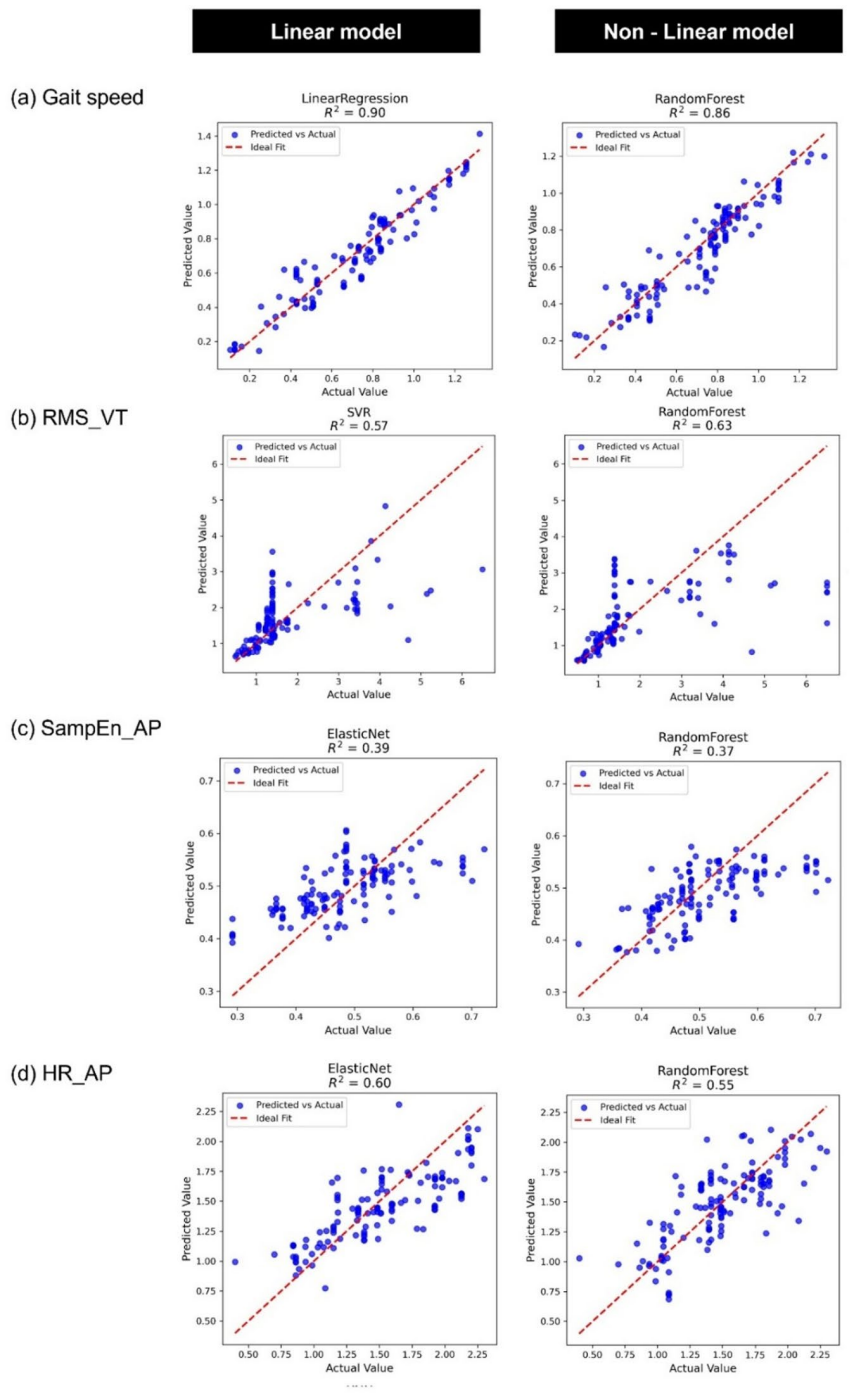
Scatter plot. Scatter plots of predicted versus actual values for four outcome variables using the best-performing linear and non-linear regression models. Each panel shows the prediction performance for one outcome variable: (**a**) Gait speed, (**b**) RMS_VT, (**c**) SampEn_AP, and (**d**) HR_AP. RMS, Root mean square; VT, Vertical; Samp_En, Sample entropy; AP, Anterior-posterior; HR, Harmonic ratio.

The performance of supervised machine learning regression models was evaluated for predicting gait speed, RMS_VT, SampEn_AP, and HR_AP during uneven-surface walking, based on 50 repeated runs with different random seeds. Among the regression models for gait speed, Linear Regression achieved the highest R² value (0.903), while RF was the most accurate among nonlinear models (R² = 0.860) (Fig. 3a). For RMS_VT, RF outperformed all the other models (R² = 0.630), whereas SVR showed the best performance among the linear models (R² = 0.575) (Fig. 3b). In contrast, the predictive performance of SampEn_AP was limited across models, with the Elastic Net (R² = 0.393) and RF (R² = 0.373) showing relatively better performances (Fig. 3c). For HR_AP, the Elastic Net (R² = 0.607) and RF (R² = 0.584) models demonstrated moderate accuracy (Fig. 3d). Notably, RMS_VT was the only outcome in which the nonlinear models consistently outperformed linear models. A complete summary of regression performance metrics is provided in Supplementary Table S4 with corresponding scatter plots shown in Supplementary Fig. S6.

### Model interpretation

**Fig. 4:**
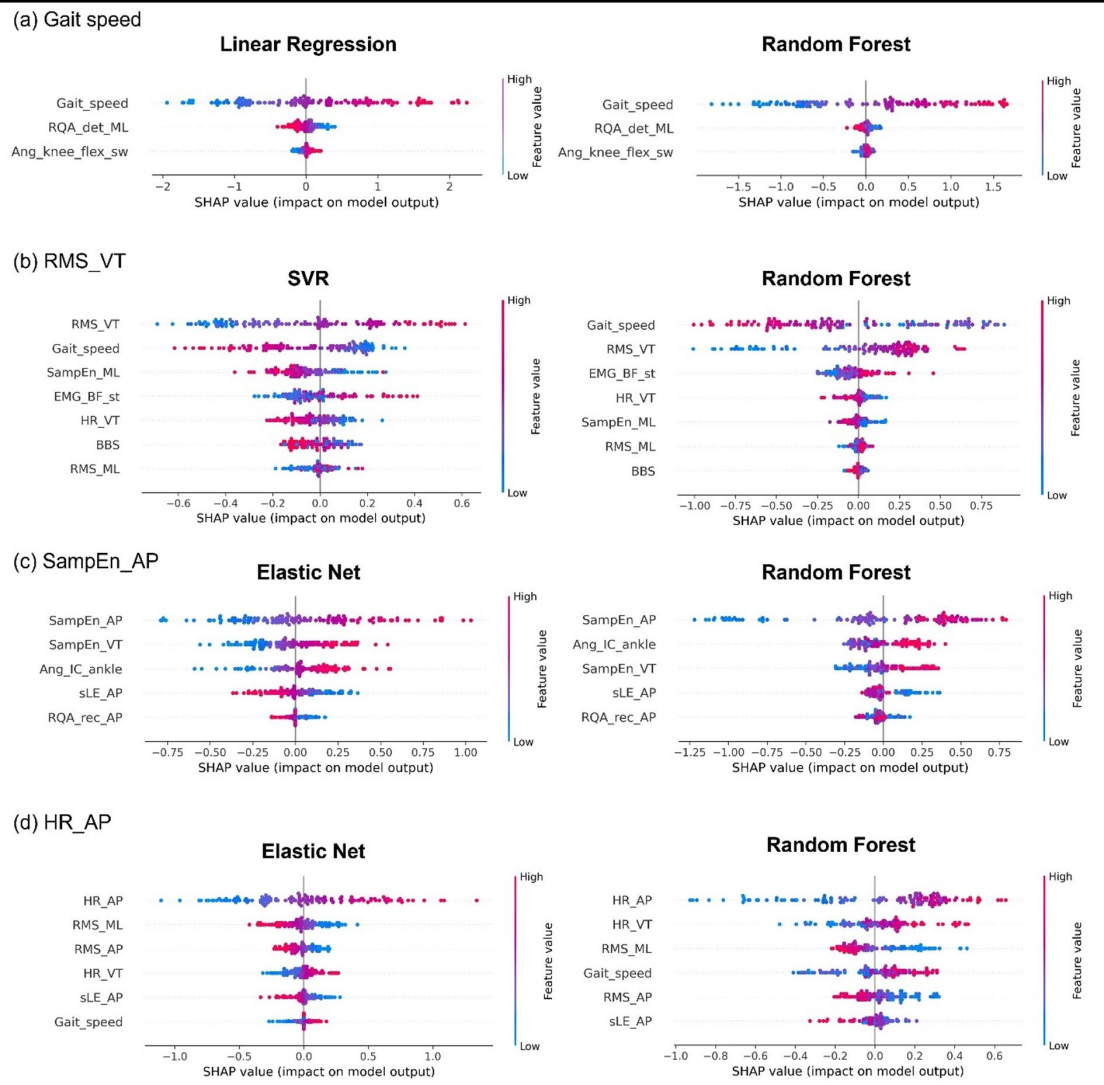
SHAP Value Plot for outcome. These SHAP value plots illustrate the even surface walking parameters that contribute to predicting each outcome on uneven surface walking. (**a**) Gait_speed; (**b**) RMS_VT; (**c**) SampEn_AP; (**d**) HR_AP. RMS, Root mean square; VT, Vertical; Samp_En, Sample entropy; AP, Anterior-posterior; HR, Harmonic ratio.

We conducted model interpretation using SHAP and PDP for the models that showed the highest performance for each target variable (Supplementary Table S4). In the prediction of uneven surface gait speed and HR_AP, both linear models (Linear Regression and Elastic Net, respectively) and nonlinear models (RF) identified their corresponding even-surface parameters (i.e., even-surface gait speed and HR_AP) as the most influential features through SHAP analysis (Fig. 4). For RMS_VT, in the nonlinear model (RF), which showed superior predictive accuracy, the even-surface gait speed was more important than the even-surface RMS_VT.

In the prediction of SampEn_AP, both the Elastic Net and RF models revealed that even-surface SampEn_AP was a key predictor. Additionally, in the RF model, the ankle dorsiflexion angle at initial contact (Ang_IC_ankle) emerged as one of the top-ranked features.

**Fig. 5:**
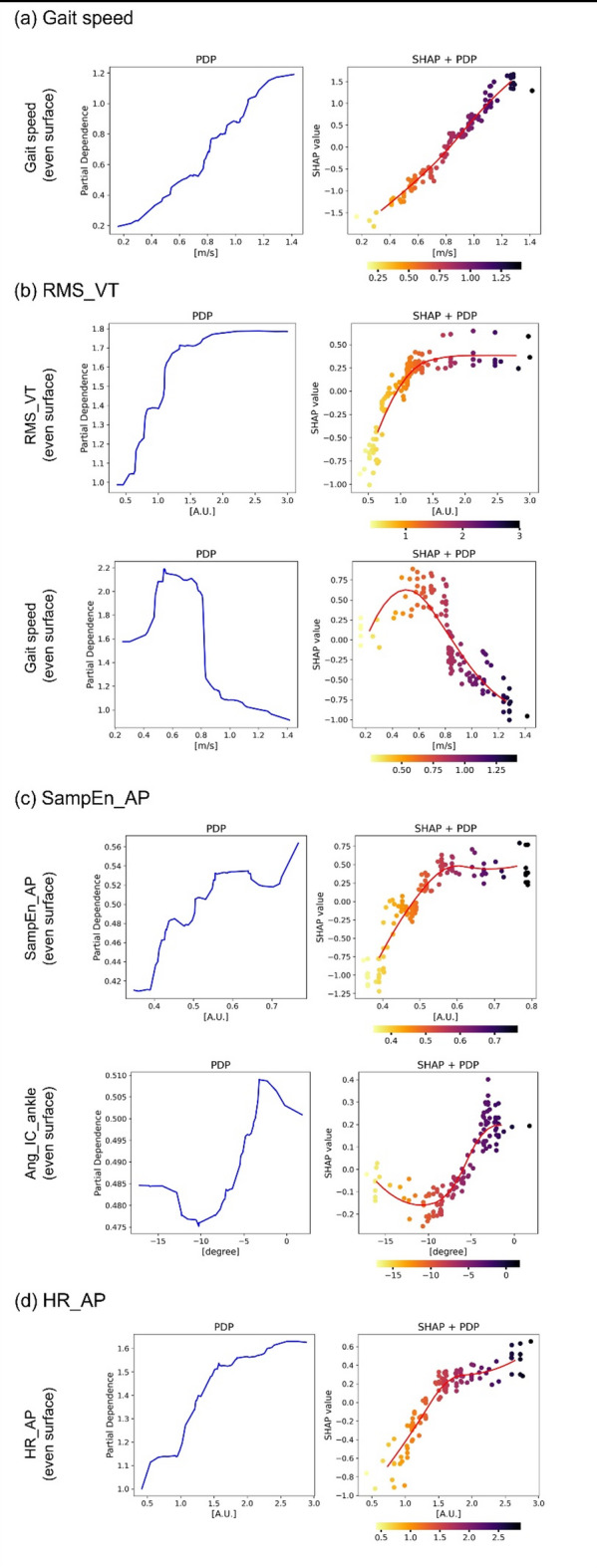
Partial dependence plot analysis and SHAP partial dependence plot analysis for gait parameters on uneven surface. Partial dependence plots (PDPs) and SHAP dependence plots for key even-surface gait features predicting uneven-surface gait outcomes using the Random Forest model. Each row corresponds to a target variable: (**a**) Gait speed, (**b**) RMS_VT, (**c**) SampEn_AP, and (**d**) HR_AP. For each variable, the left panel shows the PDP, and the right panel shows the SHAP dependence plot with individual data points colored by feature value. Plots are presented for the most influential same-name feature (top row) and an additional relevant predictor (bottom row) identified through SHAP analysis. RMS, Root mean square; VT, Vertical; Samp_En, Sample entropy; AP, Anterior-posterior; HR, Harmonic ratio.

PDP and SHAP + PDP plots were used to assess the contribution of each even-surface parameter to the prediction of uneven-surface outcomes. In the linear models, all relationships appeared approximately linear, as shown in Supplementary Fig. S7. In contrast, Fig. 5 illustrates the results from the nonlinear (RF) models, highlighting more complex relationships.

For Gait speed, participants with an even-surface walking speed below 0.8 m/s tended to show greater reductions on uneven surfaces. Similarly, the RMS_VT prediction model indicated a plateau in the RMS_VT values when the even surface walking speed was below 0.8 m/s. For SampEn_AP, both excessively large and small values of Ang_IC_ankle were associated with a higher SampEn_AP, suggesting a U-shaped dependency. As for HR_AP, the uneven-surface HR_AP plateaued when the even-surface HR_AP exceeded approximately 1.5.

## Discussion

In this study, we applied a two-step ML approach to analyze trunk acceleration during uneven-surface walking. Step 1 identified key features distinguishing PwS from HC using data augmentation and classification models. Step 2 used regression models to predict these features and the uneven-surface gait speed from the even-surface walking parameters.

Step 1 identified RMS_VT, SampEn_AP, and HR_AP as key features distinguishing PwS from HC. In regression analysis, PwS with even-surface gait speed < 0.8 m/s showed greater speed decline and elevated RMS_VT on uneven surfaces. SampEn_AP was influenced by ankle dorsiflexion at the initial contact, and HR_AP was predicted by its even surface value. These findings highlight that multidimensional, noninvasive sensor data can detect gait instability in PwS and may contribute to the advancement of individualized gait assessment aimed at supporting outdoor mobility.

Among the trunk acceleration metrics for uneven-surface walking, RMS_VT (linear), SampEn_AP, and HR_AP (nonlinear) were particularly effective in distinguishing PwS from HC. RMS_VT was highly important across the models: a significant odds ratio in LR (Table 2), high SHAP values in RF (Fig. 2), and a high VIP score in sPLS-DA (Table 3). Features consistently selected across models are considered robust digital biomarkers^33–35^. This supports prior findings of elevated RMS in PwS during uneven-surface walking⁹ and suggests that increased RMS_VT may reflect greater postural demands or an impaired response to perturbations^36^. Although acceleration-based measures can be affected by anthropometric factors such as body size and segment mass, no group differences in these characteristics were observed in our dataset, suggesting minimal confounding from morphology. For the nonlinear indices, elevated SampEn_AP contributed to PwS classification in both the LR and SVC models (Fig. 2). Although a higher sample entropy typically reflects adaptive motor control in healthy individuals^8^, it co-occurs with increased RMS in PwS. This suggests that an elevated SampEn_AP in PwS may indicate instability or irregular trunk acceleration rather than flexible control during uneven-surface walking.

A lower HR_AP was a key predictor of PwS in both the SHAP analysis of the SVC model and the VIP scores from the sPLS-DA. HR reflects waveform periodicity, and previous studies have reported that HR decreases on uneven surfaces, even in healthy individuals^4^. In PwS, HR is typically lower than that in HC^37^, consistent with our findings. Notably, HR_AP was more important than RMS in the SVC model, suggesting that disturbances in the harmonic organization or periodic structure of the trunk acceleration waveform may capture instability more sensitively than changes in waveform magnitude alone.

Gait speed during uneven-surface walking was highly predictable from even-surface parameters, with strong performance in both linear (LR, R² = 0.903) and nonlinear (RF, R² = 0.860) models (Fig. 3). SHAP analysis identified even-surface gait speed as the most influential predictor (Fig. 4), and PDP plots showed that PwS walking below 0.8 m/s exhibited greater speed reductions on uneven surfaces (Fig. 5). For RMS_VT, moderate prediction accuracy was achieved with SVR (R² = 0.575) and RF (R² = 0.630), with RF performing marginally better. SHAP analysis indicated that even-surface gait speed had a greater influence than RMS_VT itself (Fig. 4), and PDP plots showed consistently high RMS_VT in PwS walking below 0.8 m/s (Fig. 5).

These findings suggest that PwS with an even-surface gait speed below 0.8 m/s are likely to show both reduced speed and increased trunk acceleration variability (high RMS_VT) on uneven surfaces, indicating difficulty in adaptation. This aligns with prior reports of further speed reduction outdoors in slower PwS³⁸. The 0.8 m/s threshold is widely used to classify gait capacity^39,40^, and our results support its validity for predicting gait speed and stability under uneven conditions. Importantly, this threshold has long served as a functional benchmark for community ambulation^41^, and our findings quantitatively reinforce its relevance in uneven-surface contexts. IMU-based ML analysis quantitatively reinforced its clinical relevance as a mobility indicator.

The predictive accuracy of SampEn_AP was limited in both linear (Elastic Net, R² = 0.393) and nonlinear (RF, R² = 0.373) models, warranting cautious interpretation. SHAP analysis identified Ang_IC_ankle during even-surface walking as a relatively important predictor. The SHAP + PDP plots showed a U-shaped relationship, with SampEn_AP increasing when dorsiflexion was either too low or too high, indicating nonlinearity (Fig. 5). Increased dorsiflexion generally supports gait stability^42^; however, in PwS, reduced tibialis anterior (TA) activity can make ankle dorsiflexion difficult^43^. Thus, decreased dorsiflexion at the IC may lead to decreased stability on uneven surfaces. However, in cases where ankle dorsiflexion is markedly large despite reduced TA activity, it may reflect compensatory overexertion and a lack of motor control flexibility^44^. Paradoxically, these conditions may result in decreased stability on uneven surfaces. Given the modest accuracy of the SampEn_AP model, this interpretation should be viewed cautiously. This limited performance may be partly explained by characteristics of entropy measures—SampEn is inherently sensitive to non-stationarity and noise in gait signals. In addition, the analysis intentionally used ten gait cycles, reflecting both safety and fatigue constraints during uneven-surface walking in PwS and our aim to assess whether stability-related biomarkers can be extracted from short, clinically realistic data segments. Prior entropy-based gait studies have shown that approximately ten strides can yield stable entropy estimates^45^, and that entropy values become largely independent of signal length once the time series exceeds roughly 750 data points^46^—well below the ~ 1,180 samples contained in each segment of our study (Supplementary Table S6). Nonetheless, shorter recordings may increase estimation variability, and future work should verify SampEn-based stability using longer continuous trials.

For HR_AP during uneven-surface walking, both the linear (SVR, R² = 0.607) and nonlinear (RF, R² = 0.547) models showed moderate predictive accuracy (Fig. 3). The SHAP analysis identified even-surface HR_AP as a key predictor (Fig. 4). In the RF model, SHAP and PDP plots showed that HR_AP on uneven surfaces increased proportionally when even-surface values were low (especially < 1.5), but plateaued above ~ 1.5 (Fig. 5), suggesting a limited additional benefit. Thus, improving HR_AP during even-surface walking may enhance gait regularity on uneven surfaces, particularly in individuals with low baseline values. As HR_AP reflects trunk coordination^47^, interventions targeting trunk function may improve stability in complex environments. The identified threshold (~ 1.5) aligns with previous reports showing mean HR_AP values of ~ 2.0 in healthy individuals and < 1.5 in PwS^37^, suggesting a physiologically meaningful cutoff value. Therefore, HR_AP may serve as a practical sensor-based indicator for rehabilitation and outdoor stability monitoring.

This study is the first to use ML with SHAP and PDP analyses to clarify the differences in gait stability indicators between HC and PwS during uneven-surface walking and their associations with even-surface gait parameters. By employing multiple models, including glass boxes, black boxes, and feature-selective approaches, we enhanced the robustness of our findings. Integrating both linear and nonlinear indicators provided a more nuanced understanding of clinically described “reduced stability.” Linking even-surface metrics to instability on uneven surfaces supports the translation of laboratory-based gait data into real-world contexts and provides individualized rehabilitation strategies for outdoor mobility in PwS.

This single-center study included a modest number of HC (*n* = 39), whereas a priori power analysis and previous IMU-based work suggested that approximately 100 participants per group would be desirable for robust inference. Although class imbalance was addressed using SMOTE, GAN, and ctGAN, these augmentation techniques were applied solely to stabilise model training and do not substitute for real participant data or increase statistical power. Therefore, the limited HC sample may constrain generalisability, and external validation using larger real-world datasets is warranted. Nonetheless, this study offers proof-of-concept for real-world digital biomarkers and supports future multicenter studies. Furthermore, feature selection (Boruta and LASSO) was conducted prior to train–test splitting, which may introduce a risk of information leakage because the selected feature set was informed by the full dataset, including samples later used for testing. Although this workflow is commonly seen in applied clinical machine learning studies^14,18^, the more rigorous strategy would involve performing feature selection within the training folds and applying the selected features to the held-out test data. Future studies should incorporate fold-wise feature selection to further minimise bias and enhance methodological robustness. Lastly, the predictive accuracy of SampEn_AP was limited, which may reflect several factors. Entropy-based metrics are inherently sensitive to noise and non-stationarity in gait signals, and SampEn was calculated from relatively short recordings (ten gait cycles), conditions that may increase variability. In addition, SampEn was derived only from the paretic side, whereas post-stroke gait asymmetry suggests that bilateral information may be necessary to fully capture stability characteristics. Accordingly, SampEn-based interpretations should be viewed cautiously, and future studies should verify entropy-derived stability using longer recordings and bilateral representations of gait.

This study used ML to analyze acceleration-based gait stability indices during uneven-surface walking, identifying key features that distinguish PwS from HC. The classification models achieved an accuracy of over 95%, with RMS_VT, SampEn_AP, and HR_AP as the key discriminators. In regression, PwS with even-surface gait speed < 0.8 m/s showed slower speeds and higher RMS_VT on uneven surfaces, indicating poor adaptability. SampEn_AP was influenced by ankle dorsiflexion, and HR_AP was influenced by its even-surface value, both of which showed nonlinear patterns. These findings highlight the utility of ML-based acceleration analysis for assessing gait stability and adaptation in PwS. Future studies should validate these results in larger cohorts and inform targeted rehabilitation strategies.

## Methods

### Participants

A cross-sectional study was conducted at the authors’ institution involving 71 PwS (63.8 ± 7.4 years; stroke onset: median 62.0 days, Interquartile Range 52.0) and 39 age-matched community-dwelling HC (65.6 ± 7.4 years). The exclusion criteria were as follows: (1) inability to walk independently, even with a single cane; (2) presence of bilateral brain lesions; (3) Mini-Mental State Examination (MMSE) score below 24; (4) history of orthopedic disorders; and (5) cerebellar lesions. Written informed consent was obtained from all participants prior to enrollment. All procedures were approved by the ethics committee of the authors’ institution and were conducted in accordance with the Declaration of Helsinki.

### Experimental setup and procedures

The participants walked three round trips on a 10-meter even-surface walkway and a 10-meter uneven-surface walkway, each with 2-meter buffer zones. A physical therapist supervised all the walking tasks to ensure safety. Standardized footwear was provided by (W503, MARIANNU Co. Ltd., Japan). The use of canes was permitted if needed; however, lower limb orthoses were not allowed.

During walking, trunk acceleration was recorded using a triaxial wireless accelerometer placed at the third lumbar vertebra (L3) to evaluate stability (see in Supplementary Fig. S8). In addition, sagittal plane videos and surface EMG signals were collected simultaneously. EMG data were obtained from the paretic side in PwS and the right side in HC, targeting five lower limb muscles: the TA, soleus (SOL), rectus femoris, biceps femoris (BF), and gluteus medius (GM)^48^. Additional technical details regarding the surface design, sensor settings, and data acquisition are provided in Supplementary Table S5.

### Clinical evaluation

The severity of lower limb motor impairment was assessed using the Fugl-Meyer Assessment (FMA)^49^. Balance ability was evaluated using the Berg Balance Scale (BBS)^50^. Before the task, the participants rated their confidence in walking on an uneven surface using a Likert scale (0 = no confidence, 10 = complete confidence) based on the modified Gait Efficacy Scale^51^. Isometric knee extensor strength on the non-paretic side was measured using a handheld dynamometer and normalized to the body weight^52^.

### Gait cycle detection

Gait speed was calculated from the time required to traverse a 10-meter walkway, based on synchronized video recordings^53^. The first and last three gait cycles were excluded to eliminate the acceleration and deceleration effects. Initial contact and toe-off were identified using the anteroposterior axis of a shank-mounted accelerometer^54^ and verified using a video. Ten gait cycles were extracted for analysis.

### Gait stability analysis

All stability metrics—both linear and nonlinear—were computed using the gait cycles remaining after exclusion of the first and last three strides to avoid transient acceleration and deceleration effects. To evaluate gait stability on even and uneven surfaces, we analyzed trunk acceleration in the anterior-posterior (AP), mediolateral (ML), and vertical (VT) directions using MATLAB R2021b (MathWorks Inc., Natick, MA, USA). Linear stability was quantified using the RMS values normalized by squared gait speed^55^. Nonlinear stability metrics include the HR^28^, SampEn^45,56^, RQA^56–58^, and sLE^59–62^, capturing smoothness, irregularity, periodicity, and local dynamic stability, respectively. The detailed computation procedures are provided in Supplementary Table S6.

### Biomechanical parameters analysis

Joint angles (hip, knee, and ankle) were calculated using OpenPose (v1.7.0), a markerless motion capture system based on video recordings^63^, validated against optical motion capture^64^. Signals were low-pass filtered using a zero-lag fourth-order Butterworth filter (6 Hz)^65^ and time-normalized to 100 points per gait cycle. Peak angles were extracted from the paretic limb in the PwS group and the right limb of the HC group. The EMG signals were bandpass-filtered (20–500 Hz), mean-centered, rectified, and low-pass-filtered at 10 Hz^66^. Each signal was normalized to the individual’s maximum amplitude and averaged over 10 strides from three trials^67^. Co-contraction indices (CIs) were computed from the overlap between TA and SOL (shank) and rectus femoris–BF (thigh) activity^66^, averaged separately for the stance and swing phases across the conditions. Preprocessing was performed according to the SENIAM guidelines.

### Step 1. Stroke detection

This step aimed to identify trunk acceleration features during uneven surface walking that differentiated PwS from HC based on 19 acceleration-based stability indicators.

#### Glass box and black box models

##### Data normalization

Preprocessing is essential in ML, particularly for imbalanced datasets^68^. Outliers were detected using the Interquartile Range (IQR) and replaced with median values owing to IQR’s robustness of the IQR to extreme and non-normal data. Power transformation-normalized skewed features and class distributions were visualized to address the imbalance. After cleaning and transformation, all features were standardized using z-score normalization to account for algorithms sensitive to feature magnitude^69^.

##### Feature selection

Feature selection was performed in two steps. First, variables with pairwise correlations ≥ 0.9 were reduced to minimize multicollinearity^70^. Subsequently, both the Boruta algorithm^71^ and LASSO^72^ were applied to the data. The features selected by both methods were deemed robust and retained as key discriminative variables^73^.

##### Machine learning algorithms

The dataset with the selected features was split into training (80%) and testing (20%) sets using stratified-random sampling. To address class imbalance and enhance model stability, three data augmentation methods were applied to the training set: SMOTE, GAN, and ctGAN. To avoid data leakage, the augmented instances were not shared across the cross-validation folds. To investigate the differences in trunk acceleration features during uneven-surface walking between PwS and HC, an appropriate sample size was estimated. Prior studies have reported effect sizes (Cohen’s d = 0.65–0.75) for RMS metrics during uneven walking^9^, and assuming a moderate effect size of 0.5, a power analysis (α = 0.05, power = 0.8) indicated that 64 participants in each group would be required. Furthermore, previous IMU-based research^28^ suggested that approximately 100 participants per group would be desirable for more stable classifier training. In the present study, the final real-world sample comprised 71 PwS and 39 HC. To stabilise model training under this imbalance, data augmentation was used to increase the minority class up to 100 samples and, additionally, to generate an extended set of 1000 synthetic instances for robustness analyses. These augmented samples were used solely for model training and do not increase statistical power or substitute for real participant data.

SMOTE generates synthetic samples by interpolating existing instances of the minority class and is widely used to improve the classification performance of imbalanced datasets^74^. GAN and ctGAN are deep learning models that generate synthetic samples via adversarial training between the generator and discriminator^75^. GANs have been shown to improve deep learning performance, particularly in clinical applications with limited data^76^. ctGAN extends this by enabling conditional generation based on class labels and is optimized for tabular data^77^, showing improved predictive performance^78^. The architecture of the model is shown in Supplementary Table S7.

We developed six ML models: two glass-box models (LR and decision tree [DT]) and four black-box models (SVC, XGBoost, RF, and k-nearest neighbors [KNN]). These models were selected to capture both linear and nonlinear decision boundaries, while balancing interpretability (glass-box models) with predictive performance (black-box models). The hyperparameters were optimized using a 5-fold cross-validated grid search (Supplementary Table S8). The models were evaluated on the test set using the ROC AUC, sensitivity, specificity, F1 score, and Brier score. Taken together, this framework enabled systematic exploration of multiple augmentation–model pairings, which we considered essential for identifying robust classifier configurations rather than assuming that any single augmentation strategy or algorithm would perform best.

To obtain robust and unbiased performance estimates while minimizing overfitting, data augmentation and ML model training were repeated 50 times with different random seeds (random state = 42), and the average performance metrics were computed^79^. Based on these results, the top-performing models from both the glass and black boxes were selected for interpretation.

Model interpretation was conducted using odds ratios for LR and SHAP values^80^ for the black box models. SHAP quantifies each feature’s contribution to the model output by averaging its marginal effect across all feature combinations, thereby providing both importance and directional insight.

##### Including feature selection model

We also used sPLS-DA^21^, a sparse version of partial least squares that integrates variable selection and classification in a single step. Unlike the two-step feature selection approach, sPLS-DA simultaneously performs both tasks. As with the other models, the data were z-score-normalized beforehand. Model accuracy was evaluated using the ROC AUC, and interpretation was based on the VIP score^81^.

### Step 2. stroke uneven prediction

Demographics, physical function, and even-surface gait parameters, including speed, trunk acceleration, paretic limb EMG, and joint kinematics, were used as input features to develop ML regression models for predicting key stability indicators and gait speed during uneven-surface walking, as identified in step 1.

### Data normalization

Even surface parameters for PwS were preprocessed as in Step 1: outliers (IQR-based) were replaced with medians, and then power transformation and z-score normalization were applied.

### Feature selection

Feature selection was performed in two steps. First, variables with pairwise correlations ≥ 0.9 were reduced to minimize multicollinearity^70^. Subsequently, both the Boruta algorithm^71^ and LASSO^72^ were applied to the data. The features selected by both methods were deemed robust and retained as key discriminative variables^82^.

### Machine learning algorithm

After feature selection, the dataset was divided into training (80%) and testing (20%) sets using stratified random sampling. Six ML models were developed: three linear (Linear Regression, SVR, and Elastic Net) and three nonlinear (RF, XGBoost, and KNN) models. The hyperparameters were optimized using a grid search with 5-fold cross-validation (Supplementary Table S9). The model performance on the test set was evaluated using R², root mean squared error (RMSE), mean absolute error (MAE), and mean squared error (MSE). To ensure robustness, reduce reporting bias, and prevent overfitting, model training was repeated 50 times with different random seeds (random state = 42), and the average metrics were reported^79^. Based on the overall performance, the best model for each group (linear and nonlinear) was selected. SHAP, PDP, and SHAP + PDP plots were used to interpret the individual feature contributions and their effects on the predictions. A visual summary of the entire two-step workflow is provided in Supplementary Fig. S9 to enhance clarity and overall understanding of the analytic pipeline.

### Statistical analysis

Age, height, weight, and BMI were compared between PwS and HC groups using Welch’s t-tests, and sex distribution was examined using Fisher’s exact test. Statistical analyses were performed using R software (version 4.1.2), with a significance level set at *p* < 0.05. All ML–related analyses were conducted using Python version 3.12.7 (with the seaborn, sklearn, imblearn, TensorFlow, SDV, CTGAN, Optuna, and SHAP packages) and R version 4.3.3 (with the mixOmics, Boruta, and glmnet packages)^21^.

## Supplementary Information

Below is the link to the electronic supplementary material.

Supplementary Material 1

## Data Availability

The datasets generated and analyzed in this study are available from the corresponding author upon reasonable request.

## Code availability

The code used for the machine learning classification and regression models in this study is available in the following public GitHub repository:

https://github.com/Yasuhiro-Inui/stroke-vs-healthy-uneven-gait-ML.

https://github.com/Yasuhiro-Inui/stroke-uneven-prediciton.

## References

[1] White, J. et al. Predictors of health-related quality of life in community-dwelling stroke survivors: a cohort study. *Fam Pract.* **33**, 382–387 (2016). 26980854 10.1093/fampra/cmw011

[2] Robinson, C. A., Shumway-Cook, A., Ciol, M. A. & Kartin, D. Participation in community walking following stroke: subjective versus objective measures and the impact of personal factors. *Phys. Ther.* **91**, 1865–1876 (2011). 22003172 10.2522/ptj.20100216

[3] Hawkins, K. A., Clark, D. J., Balasubramanian, C. K. & Fox, E. J. Walking on uneven terrain in healthy adults and the implications for people after stroke. *NeuroRehabilitation* **41**, 765–774 (2017). 28946584 10.3233/NRE-172154 PMC5845824

[4] Menz, H. B., Lord, S. R. & Fitzpatrick, R. C. Acceleration patterns of the head and pelvis when walking on level and irregular surfaces. *Gait Posture*. **18**, 35–46 (2003). 12855299 10.1016/s0966-6362(02)00159-5

[5] Santuz, A., Ekizos, A., Eckardt, N., Kibele, A. & Arampatzis, A. Challenging human locomotion: stability and modular organisation in unsteady conditions. *Sci. Rep.* **8**, 2740 (2018). 29426876 10.1038/s41598-018-21018-4 PMC5807318

[6] Bruijn, S. M., Meijer, O. G., Beek, P. J. & van Dieën, J. H. Assessing the stability of human locomotion: a review of current measures. *J. R Soc. Interface*. **10**, 20120999 (2013). 23516062 10.1098/rsif.2012.0999 PMC3645408

[7] Hill, K., Ellis, P., Bernhardt, J., Maggs, P. & Hull, S. Balance and mobility outcomes for stroke patients: a comprehensive audit. *Aust J. Physiother*. **43**, 173–180 (1997). 11676685 10.1016/s0004-9514(14)60408-6

[8] Lockhart, T. E. et al. Prediction of fall risk among community-dwelling older adults using a wearable system. *Sci. Rep.* **11**, 20976 (2021). 34697377 10.1038/s41598-021-00458-5 PMC8545936

[9] Inui, Y. et al. Characteristics of uneven surface walking in stroke patients: modification in Biomechanical parameters and muscle activity. *Gait Posture*. **103**, 203–209 (2023). 37245334 10.1016/j.gaitpost.2023.05.022

[10] Xu, H., Hunt, M. E., Foreman, K. B., Zhao, J. & Merryweather, A. Gait alterations on irregular surface in people with parkinson’s disease. *Clin. Biomech.* **57**, 93–98 (2018). 10.1016/j.clinbiomech.2018.06.013 29966960

[11] Buckley, C. et al. The role of movement analysis in diagnosing and monitoring neurodegenerative conditions: insights from gait and postural control. *Brain Sci.* **9**, 34 (2019). 30736374 10.3390/brainsci9020034 PMC6406749

[12] Hummel, J. et al. Clustering approaches for gait analysis within neurological disorders: a narrative review. *Digit. Biomark.* **8**, 93 (2024). 38721018 10.1159/000538270 PMC11078540

[13] Abdollahi, M. et al. Fall risk assessment in stroke survivors: a machine learning model using detailed motion data from common clinical tests and motor-cognitive dual-tasking. *Sensors* **24**, 812 (2024). 38339529 10.3390/s24030812 PMC10857540

[14] Trabassi, D. et al. Machine learning approach to support the detection of parkinson’s disease in IMU-based gait analysis. *Sensors* **22**, 3700 (2022). 35632109 10.3390/s22103700 PMC9148133

[15] Mirelman, A. et al. Detecting sensitive mobility features for parkinson’s disease stages via machine learning. *Mov. Disord*. **36**, 2144–2155 (2021). 33955603 10.1002/mds.28631

[16] Mannini, A. et al. A machine learning framework for gait classification using inertial sensors: application to elderly, post-stroke and huntington’s disease patients. *Sensors* **16**, 134 (2016). 26805847 10.3390/s16010134 PMC4732167

[17] Liuzzi, P. et al. Machine learning-based Estimation of dynamic balance and gait adaptability in persons with neurological diseases using inertial sensors. *Sci. Rep.* **13**, 8640 (2023). 37244933 10.1038/s41598-023-35744-x PMC10224964

[18] Wang, K. et al. Interpretable prediction of 3-year all-cause mortality in patients with heart failure caused by coronary heart disease based on machine learning and SHAP. *Comput. Biol. Med.* **137**, 104813 (2021). 34481185 10.1016/j.compbiomed.2021.104813

[19] Khan, I. U. et al. A proactive attack detection for heating, ventilation, and air conditioning (HVAC) system using explainable extreme gradient boosting model (XGBoost). *Sensors* **22**, 9235 (2022). 36501938 10.3390/s22239235 PMC9740645

[20] Lee, T. et al. A brief history of artificial intelligence embryo selection: from black-box to glass-box. *Hum. Reprod.* **39**, 285–292 (2024). 38061074 10.1093/humrep/dead254 PMC11016335

[21] Lê Cao, K. A. et al. Sparse PLS discriminant analysis: biologically relevant feature selection and graphical displays for multiclass problems. *BMC Bioinform.* **12**, 253 (2011). 10.1186/1471-2105-12-253 PMC3133555 21693065

[22] Mazurowski, M. A. et al. Training neural network classifiers for medical decision making: the effects of imbalanced datasets on classification performance. *Neural Netw.* **21**, 427–436 (2008). 18272329 10.1016/j.neunet.2007.12.031 PMC2346433

[23] Ambesange, S. et al. Optimizing liver disease prediction with random forest by various data balancing techniques. *Proc IEEE Int Conf Cloud Comput Emerg Mark*. 98–102 (2020). (2020).

[24] Lopez-Nava, I. H. et al. Gait activity classification on unbalanced data from inertial sensors using shallow and deep learning. *Sensors* **20**, 4756 (2020). 32842459 10.3390/s20174756 PMC7506657

[25] Fernández, A. et al. SMOTE for learning from imbalanced data: progress and challenges, marking the 15-year anniversary. *J. Artif. Intell. Res.* **61**, 863–905 (2018).

[26] Goodfellow, I. J. et al. Generative adversarial networks. *ArXiv* **1406**, 2661 (2014).

[27] Xu, L. et al. Modeling tabular data using conditional GAN. *Adv. Neural Inf. Process. Syst.* **32**, 7335–7345 (2019).

[28] Trabassi, D. et al. Optimizing rare disease gait classification through data balancing and generative AI: insights from hereditary cerebellar ataxia. *Sensors* **24**, 3613 (2024). 38894404 10.3390/s24113613 PMC11175240

[29] Sharma, Y. et al. Factors influencing the clinical adoption of quantitative gait analysis technology with a focus on clinical efficacy and clinician perspectives: a scoping review. *Gait Posture*. **108**, 228–242 (2024). 38134709 10.1016/j.gaitpost.2023.12.003

[30] Lu, X. et al. Application of isokinetic dynamometry data in predicting gait deviation index using machine learning in stroke patients: a cross-sectional study. *Sensors* **24**, 7258 (2024). 39599035 10.3390/s24227258 PMC11598631

[31] Navita et al. Gait-based parkinson’s disease diagnosis and severity classification using force sensors and machine learning. *Sci. Rep.* **15**, 328 (2025). 39747956 10.1038/s41598-024-83357-9 PMC11696931

[32] Khan, S. et al. Predicting the governing factors for the release of colloidal phosphorus using machine learning. *Chemosphere* **362**, 142699 (2024). 38944354 10.1016/j.chemosphere.2024.142699

[33] Jeong, N. et al. Elucidating governing factors of PFAS removal by polyamide membranes using machine learning and molecular simulations. *Nat. Commun.* **15**, 10918 (2024). 39738140 10.1038/s41467-024-55320-9 PMC11686221

[34] Chen, Y. et al. Predictive modeling of arginine vasopressin deficiency after transsphenoidal pituitary adenoma resection by using multiple machine learning algorithms. *Sci. Rep.* **14**, 22210 (2024). 39333611 10.1038/s41598-024-72486-w PMC11436865

[35] Li, J. et al. SNPs and blood inflammatory marker featured machine learning for predicting the efficacy of fluorouracil-based chemotherapy in colorectal cancer. *Sci. Rep.* **14**, 27700 (2024). 39532939 10.1038/s41598-024-79036-4 PMC11557704

[36] Osaka, H. et al. Association between trunk acceleration during walking and clinically assessed balance in patients with stroke. *NeuroRehabilitation* **41**, 783–790 (2017). 29254113 10.3233/NRE-172171

[37] Iosa, M. et al. Stability and harmony of gait in patients with subacute stroke. *J Med. Biol. Eng*. **36**, 635–643 (2016). 27853414 10.1007/s40846-016-0178-0 PMC5083768

[38] Taylor, D., Stretton, C. M., Mudge, S. & Garrett, N. Does clinic-measured gait speed differ from gait speed measured in the community in people with stroke? *Clin. Rehabil*. **20**, 438–444 (2006). 16774095 10.1191/0269215506cr945oa

[39] Hosoi, Y. et al. Estimation of minimal detectable change in the 10-meter walking test for patients with stroke: a study stratified by gait speed. *Front. Neurol.* **14**, 1219505 (2023). 37538254 10.3389/fneur.2023.1219505 PMC10395330

[40] Huizenga, D. et al. Wearable gait device for stroke gait rehabilitation at home. Top. *Stroke Rehabil*. **28**, 443–455 (2021). 10.1080/10749357.2020.1834272 33261520

[41] Perry, J., Garrett, M., Gronley, J. K. & Mulroy, S. J. Classification of walking handicap in the stroke population. *Stroke* **26**, 982–989 (1995). 7762050 10.1161/01.str.26.6.982

[42] Vette, A. H. et al. The utility of normative foot floor angle data in assessing toe-walking. *Foot (Edinb)*. **37**, 65–70 (2018). 30326414 10.1016/j.foot.2018.07.003

[43] Skvortsov, D. V. et al. Typical changes in gait biomechanics in patients with subacute ischemic stroke. *Diagnostics (Basel)*. **15**, 511 (2025). 40075759 10.3390/diagnostics15050511 PMC11898933

[44] Hak, L. et al. Steps to take to enhance gait stability: the effect of Stride frequency, Stride length, and walking speed on local dynamic stability and margins of stability. *PLoS One*. **8**, e82842 (2013). 24349379 10.1371/journal.pone.0082842 PMC3862734

[45] Bisi, M. C. & Stagni, R. Complexity of human gait pattern at different ages assessed using multiscale entropy: from development to decline. *Gait Posture*. **47**, 37–42 (2016). 27264400 10.1016/j.gaitpost.2016.04.001

[46] Costa, M., Peng, C. K., Goldberger, L. A. & Hausdorff, J. M. Multiscale entropy analysis of human gait dynamics. *Phys. A*. **330**, 53–60 (2003). 10.1016/j.physa.2003.08.022 PMC9070539 35518362

[47] Isho, T. & Usuda, S. Association of trunk control with mobility performance and accelerometry-based gait characteristics in hemiparetic patients with subacute stroke. *Gait Posture*. **44**, 89–93 (2016). 27004638 10.1016/j.gaitpost.2015.11.011

[48] Clark, D. J. et al. Merging of healthy motor modules predicts reduced locomotor performance and muscle coordination complexity post-stroke. *J. Neurophysiol.* **103**, 844–857 (2010). 20007501 10.1152/jn.00825.2009 PMC2822696

[49] Fugl-Meyer, A. R., Jääskö, L., Leyman, I., Olsson, S. & Steglind, S. The post-stroke hemiplegic patient. 1. A method for evaluation of physical performance. *Scand. J. Rehabil Med.* **7**, 13–31 (1975). 1135616

[50] Berg, K. O., Wood-Dauphinee, S. L., Williams, J. I. & Maki, B. Measuring balance in the elderly: validation of an instrument. Can. *J. Public. Health*. **83**, S7–S11 (1992). 1468055

[51] Newell, A. M. et al. The modified gait efficacy scale: Establishing the psychometric properties in older adults. *Phys. Ther.* **92**, 318–328 (2012). 22074940 10.2522/ptj.20110053 PMC3269773

[52] Riddle, D. L. et al. Intrasession and intersession reliability of hand-held dynamometer measurements taken on brain-damaged patients. *Phys. Ther.* **69**, 182–194 (1989). 2919189 10.1093/ptj/69.3.182

[53] Karpman, C., Lebrasseur, N. K., Depew, Z. S., Novotny, P. J. & Benzo, R. P. Measuring gait speed in the out-patient clinic: methodology and feasibility. *Respir Care*. **59**, 531–537 (2014). 23983271 10.4187/respcare.02688

[54] Kotiadis, D., Hermens, H. J. & Veltink, P. H. Inertial gait phase detection for control of a drop foot stimulator. *Med. Eng. Phys.* **32**, 287–297 (2010). 20153237 10.1016/j.medengphy.2009.10.014

[55] Mizuta, N. et al. Walking characteristics including mild motor paralysis and slow walking speed in poststroke patients. *Sci. Rep.* **10**, 68905 (2020). 10.1038/s41598-020-68905-3 PMC7366923 32678273

[56] Tamburini, P., Mazzoli, D. & Stagni, R. Towards an objective assessment of motor function in sub-acute stroke patients: relationship between clinical rating scales and instrumental gait stability indexes. *Gait Posture*. **59**, 58–64 (2018). 28988025 10.1016/j.gaitpost.2017.09.033

[57] Labini, F. S. et al. Recurrence quantification analysis of gait in normal and hypovestibular subjects. *Gait Posture*. **35**, 48–55 (2012). 21900012 10.1016/j.gaitpost.2011.08.004

[58] Riva, F. et al. Estimating fall risk with inertial sensors using gait stability measures that do not require step detection. *Gait Posture*. **38**, 170–174 (2013). 23726429 10.1016/j.gaitpost.2013.05.002

[59] Kao, P. C. et al. Dynamic instability during post-stroke hemiparetic walking. *Gait Posture*. **40**, 457–463 (2014). 24931112 10.1016/j.gaitpost.2014.05.014 PMC4251664

[60] Martinis, L. et al. Differences in trunk acceleration-derived gait indexes in stroke subjects with and without stroke-induced immunosuppression. *Sensors* **24**, 6012 (2024). 39338758 10.3390/s24186012 PMC11435490

[61] Castiglia, S. F. et al. Ability of a set of trunk inertial indexes of gait to identify gait instability and recurrent fallers in parkinson’s disease. *Sensors* **21**, 3449 (2021). 34063468 10.3390/s21103449 PMC8156709

[62] Arpan, I. et al. Local dynamic stability during long-fatiguing walks in people with multiple sclerosis. *Gait Posture*. **76**, 122–127 (2020). 31760315 10.1016/j.gaitpost.2019.10.032

[63] Cao, Z., Simon, T., Wei, S. E. & Sheikh, Y. Realtime multi-person 2D pose estimation using part affinity fields. In Proceedings of the IEEE Conference on Computer Vision and Pattern Recognition 1302–1310 (2017).

[64] Ota, M., Tateuchi, H., Hashiguchi, T. & Ichihashi, N. Verification of validity of gait analysis systems during treadmill walking and running using human pose tracking algorithm. *Gait Posture*. **85**, 290–297 (2021). 33636458 10.1016/j.gaitpost.2021.02.006

[65] Routson, R. L., Clark, D. J., Bowden, M. G., Kautz, S. A. & Neptune, R. R. The influence of locomotor rehabilitation on module quality and post-stroke hemiparetic walking performance. *Gait Posture*. **38**, 511–517 (2013). 23489952 10.1016/j.gaitpost.2013.01.020 PMC3687005

[66] Murley, G. M., Menz, H. B., Landorf, K. B. & Bird, A. R. Reliability of lower limb electromyography during overground walking: a comparison of maximal- and sub-maximal normalisation techniques. *J. Biomech.* **43**, 749–756 (2010). 19909958 10.1016/j.jbiomech.2009.10.014

[67] Van Kammen, K., Boonstra, A. M. & Van Der Woude, L. H. Differences in muscle activity and Temporal step parameters between Lokomat guided walking and treadmill walking in post-stroke hemiparetic patients and healthy walkers. *J. Neuroeng. Rehabil*. **14**, 32 (2017). 28427422 10.1186/s12984-017-0244-z PMC5397709

[68] de Werner, V., Aranda, S., dos Santos Costa, J. A., da Silva Pereira, R., Victória Barbosa, J. L. & P.R. & Imbalanced data preprocessing techniques for machine learning: a systematic mapping study. *Knowl. Inf. Syst.* **65**, 31–57 (2023). 36405957 10.1007/s10115-022-01772-8 PMC9645765

[69] Ahsan, M. M., Mahmud, M. A. P., Saha, P. K., Gupta, K. D. & Siddique, Z. Effect of data scaling methods on machine learning algorithms and model performance. *Technologies* **9**, 52 (2021).

[70] Yasin, P. et al. Machine learning-enabled prediction of prolonged length of stay in hospital after surgery for tuberculosis spondylitis patients with unbalanced data: a novel approach using explainable artificial intelligence (XAI). *Eur. J. Med. Res.* **29**, 383 (2024). 39054495 10.1186/s40001-024-01988-0 PMC11270948

[71] Kursa, M. B. & Rudnicki, W. R. Feature selection with the Boruta package. *J. Stat. Softw.* **36**, 1–13 (2010).

[72] Friedman, J., Hastie, T. & Tibshirani, R. Regularization paths for generalized linear models via coordinate descent. *J Stat. Softw*. **33**, 1–22 (2010). 20808728 PMC2929880

[73] Huang, X., Dai, Z., Wang, K. & Luo, X. Machine learning-based prediction of binge drinking among adults in the united states: analysis of the 2022 health information National trends survey. *Proc. 2024 9th Int. Conf. Math. Artif. Intell.* **2024**, 1–10 (2024). 10.1145/3670085.3670090 PMC11745038 39834720

[74] Chawla, N. V., Bowyer, K. W., Hall, L. O. & Kegelmeyer, W. P. SMOTE: synthetic minority over-sampling technique. *J. Artif. Intell. Res.* **16**, 321–357 (2002).

[75] Goodfellow, I. J. et al. Generative adversarial networks. *Sci. Robot*. **3**, 2672–2680 (2014).

[76] Ramesh, V. & Bilal, E. Detecting motor symptom fluctuations in parkinson’s disease with generative adversarial networks. *NPJ Digit. Med.* **5**, 138 (2022). 36085350 10.1038/s41746-022-00674-x PMC9463161

[77] Xu, L., Skoularidou, M., Cuesta-Infante, A. & Veeramachaneni, K. Modeling tabular data using conditional GAN. Adv. *Neural Inf. Process. Syst* **32** (2019).

[78] Ma, H. et al. Data augmentation of a corrosion dataset for defect growth prediction of pipelines using conditional tabular generative adversarial networks. *Materials* **17**, 1142 (2024). 38473613 10.3390/ma17051142 PMC10934152

[79] Blüthgen, C. et al. Computed tomography radiomics for the prediction of thymic epithelial tumor histology, TNM stage and myasthenia Gravis. *PLoS One*. **16**, e0261401 (2021). 34928978 10.1371/journal.pone.0261401 PMC8687592

[80] Lundberg, S. M. & Lee, S. I. A unified approach to interpreting model predictions. *Adv. Neural Inf. Process. Syst*. **30**, 4766–4775 (2017).

[81] Patro, A. R. K. et al. Cytokine signature associated with disease severity in dengue. *Viruses* **11**, 34 (2019). 30626045 10.3390/v11010034 PMC6357178

[82] He, W. et al. Development and evaluation of interpretable machine learning regressors for predicting femoral neck bone mineral density in elderly men using NHANES data. *Biomol. Biomed.* **25**, 375–390 (2025). 38972052 10.17305/bb.2024.10725 PMC11734819

