# Supplementary material for: Identifying and predicting gait stability metrics in people with stroke in uneven-surface walking using machine learning
Source: Sci Rep. 2026 Jan 17;16:5618. doi: 10.1038/s41598-026-35966-9 (PMC12891470; doi:10.1038/s41598-026-35966-9)
Supplement: Supplementary file 1 — Supplementary Material 1 [file 41598_2026_35966_MOESM1_ESM.docx]

| Supplementary Table S1. Summary of gait parameters | | | | |
| --- | --- | --- | --- | --- |
| Variables | Healthy Control (N=39) |  | People with Stroke (N=71) | |
|  | Uneven |  | Uneven | even |
| **Stability** |  |  |  |  |
| RMS_AP | 0.30 (0.15) |  | 0.81 (1.52) | 0.65 (0.61) |
| RMS_ML | 0.11 (0.33) |  | 0.30 (0.46) | 0.21 (0.21) |
| RMS_VT | 0.54 (0.20) |  | 1.39 (2.40) | 1.11 (0.94) |
| HR_AP | 2.30 (0.86) |  | 1.48 (0.64) | 1.59 (0.77) |
| HR_ML | 1.77 (0.54) |  | 1.55 (0.46) | 1.65 (0.64) |
| HR_VT | 2.51 (0.62) |  | 1.76 (0.82) | 2.02 (1.16) |
| SampEn_AP | 0.38 (0.09) |  | 0.49 (0.12) | 0.50 (0.15) |
| SampEn_ML | 0.61 ± 0.10 |  | 0.57 ± 0.10 | 0.63 ± 0.10 |
| SampEn_VT | 0.39 (0.09) |  | 0.39 (0.14) | 0.40 (0.11) |
| RQA_rec_AP (%) | 2.58 (2.33) |  | 1.74 (1.48) | 1.44 (1.04) |
| RQA_rec_ML (%) | 1.43 (1.16) |  | 1.34 (1.19) | 1.01 (0.71) |
| RQA_rec_VT (%) | 3.21 (2.39) |  | 4.91 (4.57) | 3.46 (2.01) |
| RQA_det_AP (%) | 28.2 ± 5.22 |  | 31.6 ± 7.62 | 30.4 ± 6.86 |
| RQA_det_ML (%) | 25.4 ± 4.58 |  | 31.5 ± 7.36 | 30.4 ± 5.60 |
| RQA_det_VT (%) | 33.3 (3.94) |  | 37.2 (7.22) | 36.6 (5.09) |
| sLE_AP | 0.37 ± 1.85 |  | 0.58 ± 0.25 | 0.50 ± 0.22 |
| sLE_ML | 0.29 ± 0.10 |  | 0.30 ± 0.11 | 0.28 ± 0.11 |
| sLE_VT | 0.31 ± 0.13 |  | 0.36 ± 0.12 | 0.34 ± 0.15 |
| sLE_3 | 0.33 ± 0.10 |  | 0.34 ± 0.11 | 0.38 ± 0.13 |
| **Gait parameters** |  |  |  |  |
| **Gait speed (m/s)** |  |  | 0.71 ± 0.29 | 0.83 ± 0.26 |
| **Kinematics (degree)** |  |  |  |  |
| Stance phase |  |  |  |  |
| Hip extension |  |  | 9.1 (9.7) | 11.1 (10.9) |
| Knee flexion |  |  | 29.2 ± 8.4 | 30.4 ± 8.1 |
| Ankle dorsiflexion |  |  | 4.1 ± 4.4 | 6.5 ± 4.3 |
| IC Ankle dorsiflexion |  |  | -8.3 ± 5.8 | -7.1 (6.6) |
| Swing phase |  |  |  |  |
| Hip flexion |  |  | 34.2 (5.6) | 28.2 ± 3.9 |
| Knee flexion |  |  | 67.2 (17.0) | 60.3 (12.5) |
| Ankle dorsiflexion |  |  | 0.24 ± 4.8 | -0.38 ± 5.2 |
| **Electromyography (%)** |  |  |  |  |
| Stance phase |  |  |  |  |
| Tibialis anterior |  |  | 23.3 (15.0) | 24.1 (13.7) |
| Soleus |  |  | 26.0 (17.4) | 32.4 (12.3) |
| Rectus femoris |  |  | 18.9 (14.3) | 22.1 (15.7) |
| Biceps femoris |  |  | 21.5 (11.2) | 19.3 (13.2) |
| Gluteus medius |  |  | 27.1 ± 10.5 | 28.9 ± 10.1 |
| Swing phase |  |  |  |  |
| Tibialis anterior |  |  | 21.2 (14.0) | 20.1 (14.6) |
| Soleus |  |  | 9.0 (8.9) | 7.9 (8.7) |
| Rectus femoris |  |  | 13.2 (14.1) | 13.4 (13.3) |
| Biceps femoris |  |  | 20.0 (13.0) | 19.8 (13.3) |
| Gluteus medius |  |  | 15.7 (10.2) | 15.1 (13.6) |
| **Co-contraction (%)** |  |  |  |  |
| Stance phase |  |  |  |  |
| shank |  |  | 59.1 (21.5) | 56.0 (25.2) |
| thigh |  |  | 63.6 (14.9) | 67.2 (15.7) |
| Swing phase |  |  |  |  |
| shank |  |  | 58.2 (27.8) | 56.9 (23.3) |
| thigh |  |  | 56.0 (22.8) | 58.4 (18.8) |
| Stability metrics during uneven-surface walking in healthy controls (HC) and people with stroke (PwS), along with walking parameters during even-surface walking in PwS. For reference, parameters during uneven-surface walking in PwS are also presented, although they were not included in the main analyses. RMS, root mean square; HR, harmonic ratio; SampEn, sample entropy; RQA_rec, %recurrence in recurrence quantification analysis; RQA_det, %determinism in recurrence quantification analysis; sLE, short-term maximum Lyapunov exponent; AP, ML, VT, anterior-posterior, mediolateral, and vertical direction of the acceleration signal, respectively. | | | | |

| Supplementary Table S2. Combinations of Data Augmentation and ML Models by Overall Classification Performance | | | | | | |
| --- | --- | --- | --- | --- | --- | --- |
| Dataset | Model | ROC_AUC | Sensitivity | Specificity | F1_Score | Brier_Score |
| gan1000 | RF | 0.960 (0.04) | 0.854 (0.07) | 0.932 (0.10) | 0.902 (0.05) | 0.087 (0.04) |
| smote1000 | RF | 0.958 (0.04) | 0.896 (0.08) | 0.807 (0.12) | 0.893 (0.06) | 0.087 (0.04) |
| smote200 | RF | 0.958 (0.04) | 0.887 (0.08) | 0.812 (0.11) | 0.889 (0.06) | 0.086 (0.04) |
| ctgan200 | LR | 0.957 (0.04) | 0.919 (0.06) | 0.812 (0.12) | 0.907 (0.05) | 0.090 (0.05) |
| ctgan1000 | LR | 0.957 (0.04) | 0.904 (0.07) | 0.815 (0.12) | 0.899 (0.05) | 0.097 (0.05) |
| gan200 | RF | 0.955 (0.04) | 0.840 (0.08) | 0.920 (0.11) | 0.890 (0.06) | 0.090 (0.04) |
| gan200 | LR | 0.954 (0.05) | 0.877 (0.07) | 0.835 (0.11) | 0.889 (0.06) | 0.091 (0.04) |
| smote200 | LR | 0.953 (0.05) | 0.904 (0.08) | 0.833 (0.12) | 0.903 (0.05) | 0.096 (0.05) |
| gan1000 | LR | 0.952 (0.05) | 0.884 (0.07) | 0.830 (0.11) | 0.892 (0.05) | 0.100 (0.05) |
| ctgan200 | RF | 0.952 (0.04) | 0.884 (0.08) | 0.828 (0.12) | 0.891 (0.06) | 0.088 (0.04) |
| smote1000 | LR | 0.952 (0.05) | 0.896 (0.09) | 0.843 (0.12) | 0.901 (0.06) | 0.103 (0.06) |
| ctgan1000 | SVC | 0.951 (0.04) | 0.897 (0.07) | 0.820 (0.13) | 0.897 (0.05) | 0.100 (0.05) |
| gan200 | SVC | 0.949 (0.05) | 0.873 (0.08) | 0.845 (0.11) | 0.889 (0.06) | 0.108 (0.05) |
| ctgan1000 | RF | 0.948 (0.05) | 0.883 (0.08) | 0.853 (0.11) | 0.897 (0.05) | 0.093 (0.04) |
| ctgan200 | SVC | 0.947 (0.05) | 0.919 (0.07) | 0.815 (0.12) | 0.907 (0.05) | 0.094 (0.05) |
| gan1000 | SVC | 0.944 (0.05) | 0.881 (0.07) | 0.818 (0.11) | 0.887 (0.05) | 0.119 (0.06) |
| ctgan1000 | XGBoost | 0.942 (0.05) | 0.886 (0.07) | 0.838 (0.12) | 0.895 (0.05) | 0.117 (0.06) |
| smote1000 | XGBoost | 0.942 (0.05) | 0.887 (0.08) | 0.818 (0.11) | 0.890 (0.06) | 0.126 (0.07) |
| ctgan200 | KNN | 0.941 (0.06) | 0.901 (0.08) | 0.830 (0.13) | 0.902 (0.06) | 0.086 (0.05) |
| ctgan200 | XGBoost | 0.934 (0.06) | 0.883 (0.07) | 0.807 (0.12) | 0.886 (0.06) | 0.124 (0.06) |
| smote200 | XGBoost | 0.924 (0.07) | 0.870 (0.10) | 0.790 (0.11) | 0.872 (0.07) | 0.142 (0.08) |
| smote200 | SVC | 0.923 (0.05) | 0.914 (0.07) | 0.787 (0.13) | 0.898 (0.05) | 0.108 (0.05) |
| ctgan1000 | KNN | 0.923 (0.06) | 0.877 (0.09) | 0.843 (0.12) | 0.891 (0.06) | 0.101 (0.05) |
| smote1000 | SVC | 0.909 (0.05) | 0.909 (0.08) | 0.787 (0.12) | 0.895 (0.05) | 0.118 (0.05) |
| gan200 | XGBoost | 0.903 (0.06) | 0.829 (0.08) | 0.943 (0.11) | 0.889 (0.05) | 0.123 (0.05) |
| smote200 | KNN | 0.903 (0.07) | 0.890 (0.08) | 0.845 (0.12) | 0.899 (0.06) | 0.105 (0.06) |
| gan200 | DT | 0.882 (0.06) | 0.830 (0.08) | 0.935 (0.11) | 0.888 (0.05) | 0.132 (0.06) |
| gan1000 | XGBoost | 0.878 (0.06) | 0.813 (0.09) | 0.953 (0.09) | 0.881 (0.06) | 0.123 (0.04) |
| gan1000 | DT | 0.877 (0.06) | 0.814 (0.09) | 0.940 (0.10) | 0.879 (0.06) | 0.140 (0.06) |
| gan1000 | KNN | 0.873 (0.07) | 0.920 (0.07) | 0.825 (0.11) | 0.911 (0.05) | 0.114 (0.06) |
| smote1000 | KNN | 0.868 (0.07) | 0.879 (0.09) | 0.838 (0.12) | 0.890 (0.06) | 0.129 (0.07) |
| gan200 | KNN | 0.867 (0.08) | 0.890 (0.08) | 0.840 (0.11) | 0.898 (0.06) | 0.127 (0.07) |
| ctgan1000 | DT | 0.843 (0.09) | 0.873 (0.09) | 0.812 (0.13) | 0.881 (0.07) | 0.148 (0.08) |
| smote1000 | DT | 0.831 (0.09) | 0.861 (0.11) | 0.800 (0.12) | 0.869 (0.08) | 0.161 (0.09) |
| ctgan200 | DT | 0.829 (0.08) | 0.850 (0.10) | 0.807 (0.12) | 0.865 (0.07) | 0.165 (0.08) |
| smote200 | DT | 0.811 (0.09) | 0.844 (0.10) | 0.777 (0.13) | 0.855 (0.07) | 0.180 (0.08) |
| Values are presented as mean (standard deviation) across 50 repetitions of training and testing.DT, Decision Tree; KNN, k-Nearest Neighbors; LR, Logistic Regression; RF, Random Forest; SVC, Support Vector Classifier. | | | | | | |

| Supplementary Table S3. Final selected even surface gait variables | | | | | | | | |
| --- | --- | --- | --- | --- | --- | --- | --- | --- |
| **Uneven surface  stability valuables** |  | Gait_speed |  | RMS_VT |  | SampEn_AP |  | HR_AP |
| **Even surface  gait parameters** |  | Gait_speed |  | RMS_VT |  | SampEn_AP |  | HR_AP |
|  |  | Ang_knee_flex_sw |  | BBS |  | SampEn_VT |  | Gait_speed |
|  |  | RQA_det_ML |  | Gait_speed |  | RQA_rec_AP |  | RMS_AP |
|  |  |  |  | RMS_ML |  | sLE_AP |  | RMS_ML |
|  |  |  |  | HR_VT |  | Ang_IC_ankle |  | HR_VT |
|  |  |  |  | SampEn_ML |  |  |  | sLE_AP |
|  |  |  |  | EMG_BF_st |  |  |  |  |
| Ang, angle; sw, swing; RQA_det, %determinism in recurrence quantification analysis; RMS, root mean square; BBS, Berg Balance Scale; HR, harmonic ratio; SampEn, sample entropy; EMG, electorymyography; RQA_rec, %recurrence in recurrence quantification analysis; sLE, short-term maximum Lyapunov exponent; IC, Initial Contact; st, stance phase; AP, ML, VT, anterior-posterior, mediolateral, and vertical direction of the acceleration signal, respectively. | | | | | | | | |

| Supplementary Table S4. Performance results of different regression models. | | | | | | | | | | |  |
| --- | --- | --- | --- | --- | --- | --- | --- | --- | --- | --- | --- |
| **Gait_speed** | | | | |  | **RMS_VT** | | | | |  |
| Model | R^2^ | RMSE | MAE | MSE |  | Model | R^2^ | RMSE | MAE | MSE |  |
|  | Mean(SD) | | | |  |  | Mean(SD) | | | |  |
| Linear  Regression | 0.903 (0.02) | 0.302 (0.02) | 0.236 (0.02) | 0.091 (0.01) |  | RF | 0.630 (0.08) | 0.600 (0.06) | 0.440 (0.03) | 0.364 (0.07) |  |
| SVR | 0.901 (0.02) | 0.304 (0.02) | 0.238 (0.02) | 0.093 (0.01) |  | XG Boost | 0.587 (0.08) | 0.635 (0.07) | 0.461 (0.04) | 0.408 (0.08) |  |
| Elastic Net | 0.894 (0.02) | 0.316 (0.02) | 0.259 (0.02) | 0.101 (0.01) |  | KNN | 0.581 (0.08) | 0.640 (0.06) | 0.511 (0.05) | 0.412 (0.07) |  |
| RF | 0.860 (0.02) | 0.363 (0.03) | 0.289 (0.02) | 0.133 (0.02) |  | SVR | 0.575 (0.14) | 0.642 (0.11) | 0.481 (0.05) | 0.425 (0.16) |  |
| XG Boost | 0.840 (0.02) | 0.389 (0.03) | 0.308 (0.02) | 0.152 (0.02) |  | Elastic Net | 0.491 (0.11) | 0.704 (0.07) | 0.525 (0.04) | 0.500 (0.10) |  |
| KNN | 0.761 (0.03) | 0.477 (0.04) | 0.385 (0.04) | 0.229 (0.04) |  | Linear  Regression | 0.418 (0.16) | 0.749 (0.09) | 0.536 (0.04) | 0.568 (0.13) |  |
|  |  |  |  |  |  |  |  |  |  |  |  |
| **SampEn_AP** | | | | |  | **HR_AP** | | | | |  |
| Model | R^2^ | RMSE | MAE | MSE |  | Model | R^2^ | RMSE | MAE | MSE |  |
|  | Mean(SD) | | | |  |  | Mean(SD) | | | |  |
| Elastic Net | 0.393 (0.09) | 0.773 (0.06) | 0.640 (0.06) | 0.602 (0.10) |  | ElasticNet | 0.607 (0.06) | 0.614 (0.05) | 0.472 (0.04) | 0.379 (0.06) |  |
| Linear  Regression | 0.384 (0.12) | 0.775 (0.05) | 0.636 (0.05) | 0.604 (0.09) |  | SVR | 0.603 (0.06) | 0.618 (0.06) | 0.476 (0.04) | 0.385 (0.08) |  |
| RF | 0.373 (0.11) | 0.784 (0.06) | 0.633 (0.05) | 0.618 (0.10) |  | Linear  Regression | 0.584 (0.07) | 0.630 (0.05) | 0.479 (0.04) | 0.400 (0.07) |  |
| SVR | 0.372 (0.11) | 0.786 (0.07) | 0.632 (0.07) | 0.622 (0.11) |  | RF | 0.547 (0.06) | 0.659 (0.05) | 0.511 (0.04) | 0.437 (0.06) |  |
| KNN | 0.351 (0.07) | 0.800 (0.06) | 0.654 (0.06) | 0.645 (0.11) |  | KNN | 0.526 (0.06) | 0.675 (0.04) | 0.536 (0.04) | 0.457 (0.06) |  |
| XG Boost | 0.276 (0.11) | 0.844 (0.07) | 0.670 (0.06) | 0.717 (0.12) |  | XG Boost | 0.509 (0.06) | 0.687 (0.06) | 0.536 (0.05) | 0.476 (0.08) |  |
| RF, Random Forest; SVR, Support Vector Regression; KNN, k-Nearest Neighbors | | | | | | | | | | |  |
|  |  |  |  |  |  |  |  |  |  |  |  |

| Supplementary Table S5. Experimental Setup Details | | |  |
| --- | --- | --- | --- |
| **Uneven surface construction** | | | |
| Component | | Specification | |
| Base layer | | 1 cm EVA foam sheet | |
| Uneven elements | | Randomly placed 4×4 cm and 8×8 cm EVA blocks  (1 cm thick) | |
| Layering | | 1–2 stacked layers | |
| Covering | | 5 mm pile artificial turf | |
| Walkway size | | 10 m length + 2 m buffer zones | |
| **Sensor Setup and Data Acquisition** | | | |
| Device | Placement | Sampling Rate | Notes |
| Tri-axial accelerometer | L3 (trunk) | 148 Hz | Wireless, stability metrics |
| Surface EMG | PwS: paretic side HC: right side | 1926 Hz | TA, SOL, RF, BF, GM |
| Video camera | Sagittal plane | 60 Hz | For gait phase identification |
| Synchronization system | – | – | Delsys Trigno, includes tri-axial accelerometers |
| Electrode preparation | – | – | Skin cleaned with alcohol before placement |
| EVA, Ethylene-Vinyl Acetate; EMG, Electromyography; TA, Tibialis Anterior; SOL, Soleus; RF, Rectus Femoris; BF, Biceps Femoris; GM, Gluteus Medius | | | |

| Supplementary Table S6. Detailed Computation Methods for Gait Stability Analysis Metric | |
| --- | --- |
| Metric | Computation Summary |
| Root Mean Square  (RMS) | Trunk acceleration signals in AP, ML, and VT directions were mean-centered and low-pass filtered at 20 Hz. RMS was computed for each axis and normalized by the square of gait speed. |
| Harmonic Ratio  (HR) | Each acceleration signal was time-normalized to 100 points per stride and transformed using a discrete Fourier transform. HR_AP and HR_VT were calculated as the ratio of the amplitudes of the first 10 even harmonics to the odd harmonics, while HR_ML was calculated as the ratio of odd to even harmonics. Signals were filtered at 20 Hz. |
| Sample Entropy  (SampEn) | Sample entropy was calculated using acceleration time series resampled to 100 Hz to assess signal regularity, with fixed parameters: m = 2 and r = 0.2. On average, each stride contained approximately 118 samples, yielding ~1180 data points per ten-stride analysis segment. |
| Recurrence Quantification Analysis  (RQA) | Recurrence plots were generated using embedding dimensions m = 2–10 and nearest-neighbor estimation. The optimal dimension m = 5 was selected using the false nearest neighbor method. Time delay τ = 10 was determined by the first minimum of average mutual information. RQA metrics included RQA_rec and RQA_det. |
| Short-term Lyapunov Exponent  (sLE) | One-dimensional time series were reconstructed into multi-dimensional state space by time-delayed embedding. Time delay τ was determined using the first minimum of Average Mutual Information, and embedding dimension m was selected using the false nearest neighbor method (max m = 10). Additionally, a combined 9-dimensional state space was constructed using the three acceleration axes and their delayed copies, with τ set to 25% of the average stride duration. For each point in the state space, its nearest neighbor was tracked over time to obtain divergence curves. The logarithm of the average divergence curve was calculated, and the slope between 0 and 0.5 strides was used as the estimate of local dynamic stability (sLE_AP, sLE_ML, sLE_VT, sLE_3). |
| AP, Anterior-posterior; ML, Mediolateral; VT, Vertical. | |

| Supplementary Table S7. Architecture and Configuration of GAN and ctGAN Models | | |
| --- | --- | --- |
| **Component** | **Configuration / Layer Description** | **Notes** |
| **GAN** | | |
| **Generator** | Input (latent_dim = 10) | Latent vector sampled from N(0,1) |
|  | Dense(128, activation='relu', Glorot Uniform) |  |
|  | Dense(256, activation='relu', Glorot Uniform) |  |
|  | Dense(output_dim, activation='linear', Glorot Uniform) | Outputs synthetic feature vector |
| **Discriminator** | Input (feature_dim) | Input real or synthetic feature vector |
|  | Dense(256, activation='relu', Glorot Uniform) |  |
|  | Dense(128, activation='relu') → Dense(1, activation='sigmoid') | Binary classification (real vs fake) |
| **Optimization** | Loss: BinaryCrossentropy; Optimizer: Adam (lr = 1e-4) | Applied separately to generator & discriminator |
| **Training** | Epochs = 5000, Batch size = 32 | No early stopping used |
| **ctGAN** | | |
| **Model** | Epochs = 500; Library = ctgan (SDV package) | Fitted separately for HC and PwS |
| **Sampling** | Sample until 100 or 500 samples/class with deduplication | Ensures balanced dataset with exact sample count |
| **Preprocessing** | LabelEncoder used to encode class (HC=0, PwS=1) | Class column converted back after generation |
| **Output** | 8 gait features + Class label; stored in ctgan_data_200/1000 | Ready-to-use format for classification tasks |

| Supplementary Table S8. Summary of Optimal Hyperparameters and Search Ranges in STEP1 | | | | |
| --- | --- | --- | --- | --- |
| Dataset | Model | Parameter optimization range | Best Parameter | Frequency |
| smote200 | DT | max_depth: [10, 20, None] | {'max_depth': 10} | 50 |
| smote200 | KNN | n_neighbors: [3, 5, 7] | {'n_neighbors': 3} | 24 |
| smote200 | LR | C: [0.1, 1, 10] | {'C': 1} | 22 |
| smote200 | RF | n_estimators: [100, 200], max_depth: [10, 20, None] | {'max_depth': 10, 'n_estimators': 200} | 28 |
| smote200 | SVC | C: [0.1, 1, 10], kernel: ['linear', 'rbf'] | {'C': 10, 'kernel': 'rbf'} | 34 |
| smote200 | XGBoost | learning_rate: [0.01, 0.1], n_estimators: [100, 200] | {'learning_rate': 0.1, 'n_estimators': 200} | 31 |
| smote1000 | DT | max_depth: [10, 20, None] | {'max_depth': 10} | 50 |
| smote1000 | KNN | n_neighbors: [3, 5, 7] | {'n_neighbors': 3} | 45 |
| smote1000 | LR | C: [0.1, 1, 10] | {'C': 10} | 45 |
| smote1000 | RF | n_estimators: [100, 200], max_depth: [10, 20, None] | {'max_depth': 10, 'n_estimators': 100} | 24 |
| smote1000 | SVC | C: [0.1, 1, 10], kernel: ['linear', 'rbf'] | {'C': 10, 'kernel': 'rbf'} | 36 |
| smote1000 | XGBoost | learning_rate: [0.01, 0.1], n_estimators: [100, 200] | {'learning_rate': 0.1, 'n_estimators': 200} | 39 |
| gan200 | DT | max_depth: [10, 20, None] | {'max_depth': 10} | 50 |
| gan200 | KNN | n_neighbors: [3, 5, 7] | {'n_neighbors': 3} | 50 |
| gan200 | LR | C: [0.1, 1, 10] | {'C': 0.1} | 50 |
| gan200 | RF | n_estimators: [100, 200], max_depth: [10, 20, None] | {'max_depth': 10, 'n_estimators': 100} | 50 |
| gan200 | SVC | C: [0.1, 1, 10], kernel: ['linear', 'rbf'] | {'C': 0.1, 'kernel': 'linear'} | 50 |
| gan200 | XGBoost | learning_rate: [0.01, 0.1], n_estimators: [100, 200] | {'learning_rate': 0.01, 'n_estimators': 100} | 30 |
| gan1000 | DT | max_depth: [10, 20, None] | {'max_depth': 10} | 50 |
| gan1000 | KNN | n_neighbors: [3, 5, 7] | {'n_neighbors': 3} | 50 |
| gan1000 | LR | C: [0.1, 1, 10] | {'C': 0.1} | 50 |
| gan1000 | RF | n_estimators: [100, 200], max_depth: [10, 20, None] | {'max_depth': 10, 'n_estimators': 100} | 50 |
| gan1000 | SVC | C: [0.1, 1, 10], kernel: ['linear', 'rbf'] | {'C': 0.1, 'kernel': 'linear'} | 50 |
| gan1000 | XGBoost | learning_rate: [0.01, 0.1], n_estimators: [100, 200] | {'learning_rate': 0.01, 'n_estimators': 100} | 44 |
| ctgan200 | DT | max_depth: [10, 20, None] | {'max_depth': 10} | 50 |
| ctgan200 | KNN | n_neighbors: [3, 5, 7] | {'n_neighbors': 7} | 38 |
| ctgan200 | LR | C: [0.1, 1, 10] | {'C': 0.1} | 25 |
| ctgan200 | RF | n_estimators: [100, 200], max_depth: [10, 20, None] | {'max_depth': 10, 'n_estimators': 200} | 26 |
| ctgan200 | SVC | C: [0.1, 1, 10], kernel: ['linear', 'rbf'] | {'C': 0.1, 'kernel': 'rbf'} | 20 |
| ctgan200 | XGBoost | learning_rate: [0.01, 0.1], n_estimators: [100, 200] | {'learning_rate': 0.1, 'n_estimators': 200} | 33 |
| ctgan1000 | DT | max_depth: [10, 20, None] | {'max_depth': 10} | 47 |
| ctgan1000 | KNN | n_neighbors: [3, 5, 7] | {'n_neighbors': 7} | 39 |
| ctgan1000 | LR | C: [0.1, 1, 10] | {'C': 0.1} | 22 |
| ctgan1000 | RF | n_estimators: [100, 200], max_depth: [10, 20, None] | {'max_depth': 10, 'n_estimators': 200} | 24 |
| ctgan1000 | SVC | C: [0.1, 1, 10], kernel: ['linear', 'rbf'] | {'C': 0.1, 'kernel': 'rbf'} | 16 |
| ctgan1000 | XGBoost | learning_rate: [0.01, 0.1], n_estimators: [100, 200] | {'learning_rate': 0.1, 'n_estimators': 200} | 40 |
| DT, Decision Tree; KNN, k-Nearest Neighbors; LR, Logistic Regression; RF, Random Forest; SVC, Support Vector Classifier. | | | | |

| Supplementary Table S9. Summary of Optimal Hyperparameters and Search Ranges | | | | |
| --- | --- | --- | --- | --- |
| Objective variable  (unven surface) | Model | Parameter optimization range | Best Parameter | Frequency |
| Gait speed | ElasticNet | alpha: [0.1, 1, 10], l1_ratio: [0.2, 0.5, 0.8] | {'l1_ratio': 0.2, 'alpha': 0.1} | 35 |
|  | KNN | n_neighbors: [3, 5, 7] | {'n_neighbors': 7} | 31 |
|  | LinearRegression | No hyperparameter tuning applied | {} | 50 |
|  | RandomForest | n_estimators: [100, 200], max_depth: [10, 20, None] | {'max_depth': 10, 'n_estimators': 100} | 19 |
|  | SVR | C: [0.1, 1, 10], kernel: ['linear', 'rbf'] | {'kernel': 'linear', 'C': 1} | 27 |
|  | XGBoost | learning_rate: [0.01, 0.1], n_estimators: [100, 200] | {'learning_rate': 0.1, 'n_estimators': 100} | 31 |
| RMS_VT | ElasticNet | alpha: [0.1, 1, 10], l1_ratio: [0.2, 0.5, 0.8] | {'l1_ratio': 0.2, 'alpha': 0.1} | 47 |
|  | KNN | n_neighbors: [3, 5, 7] | {'n_neighbors': 5} | 28 |
|  | LinearRegression | No hyperparameter tuning applied | {} | 50 |
|  | RandomForest | n_estimators: [100, 200], max_depth: [10, 20, None] | {'max_depth': 10, 'n_estimators': 200} | 17 |
|  | SVR | C: [0.1, 1, 10], kernel: ['linear', 'rbf'] | {'kernel': 'rbf', 'C': 1} | 41 |
|  | XGBoost | learning_rate: [0.01, 0.1], n_estimators: [100, 200] | {'learning_rate': 0.01, 'n_estimators': 200} | 29 |
| SampEn_AP | ElasticNet | alpha: [0.1, 1, 10], l1_ratio: [0.2, 0.5, 0.8] | {'l1_ratio': 0.2, 'alpha': 0.1} | 45 |
|  | KNN | n_neighbors: [3, 5, 7] | {'n_neighbors': 7} | 27 |
|  | LinearRegression | No hyperparameter tuning applied | {} | 50 |
|  | RandomForest | n_estimators: [100, 200], max_depth: [10, 20, None] | {'max_depth': 10, 'n_estimators': 200} | 18 |
|  | SVR | C: [0.1, 1, 10], kernel: ['linear', 'rbf'] | {'kernel': 'rbf', 'C': 1} | 33 |
|  | XGBoost | learning_rate: [0.01, 0.1], n_estimators: [100, 200] | {'learning_rate': 0.01, 'n_estimators': 200} | 28 |
| HR_AP | ElasticNet | alpha: [0.1, 1, 10], l1_ratio: [0.2, 0.5, 0.8] | {'l1_ratio': 0.2, 'alpha': 0.1} | 44 |
|  | KNN | n_neighbors: [3, 5, 7] | {'n_neighbors': 7} | 29 |
|  | LinearRegression | No hyperparameter tuning applied | {} | 50 |
|  | RandomForest | n_estimators: [100, 200], max_depth: [10, 20, None] | {'max_depth': 10, 'n_estimators': 200} | 19 |
|  | SVR | C: [0.1, 1, 10], kernel: ['linear', 'rbf'] | {'C': 0.1, 'kernel': 'linear'} | 43 |
|  | XGBoost | learning_rate: [0.01, 0.1], n_estimators: [100, 200] | {'learning_rate': 0.01, 'n_estimators': 200} | 47 |
| RMS, Root mean square; VT, Vertical; Samp_En, Sample entropy; AP, Anterior-posterior; HR, Harmonic ratio. SVR, Support Vector Regression; KNN, k-Nearest Neighbors | | | | |


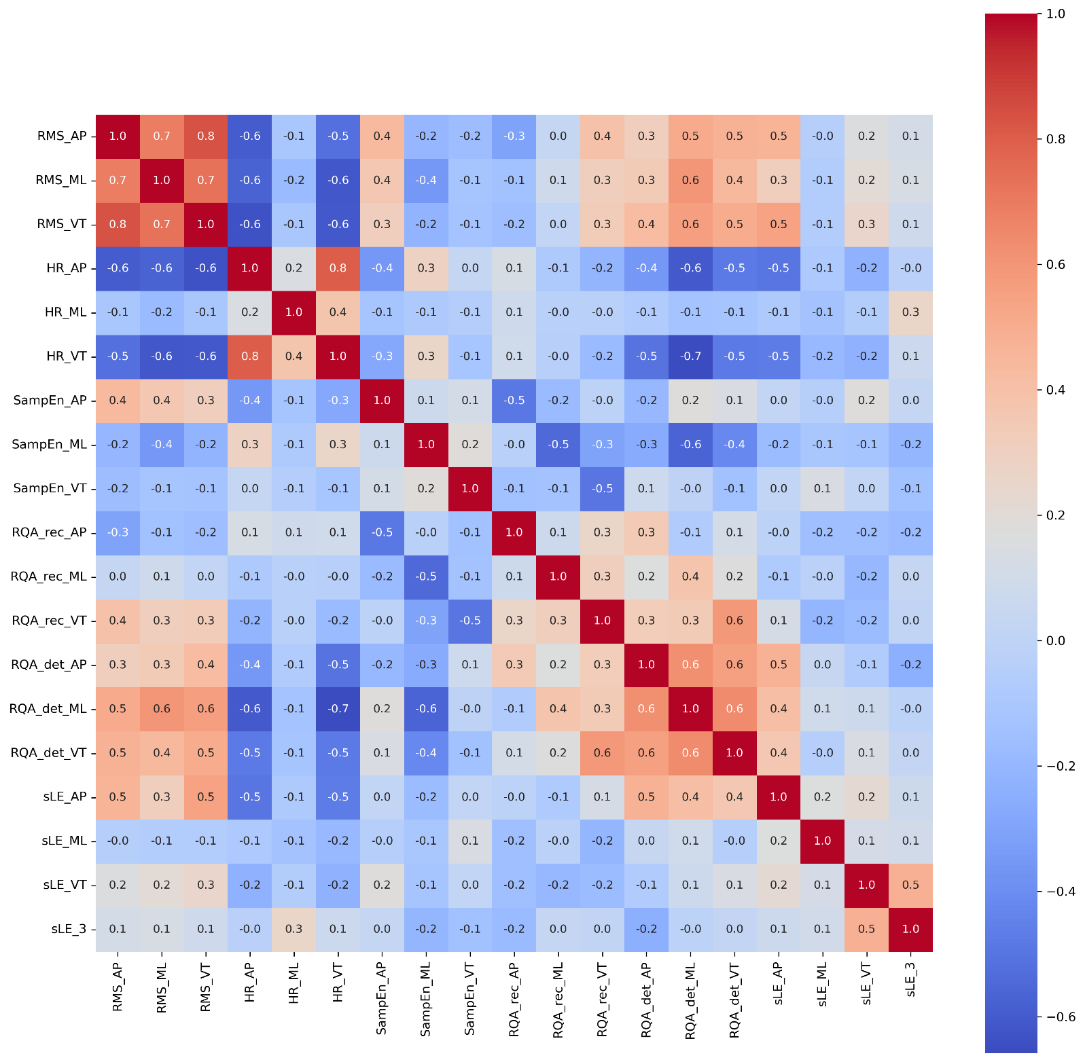


**Supplementary Fig. S1. Correlation heatmap (step1: uneven surface walking)**

The heatmap obtained using the Seaborn library's relplot function displays the correlation between initial features of the acceleration index during uneven surface walking. Each cell in the matrix represents the partial correlation between two variables, as indicated by the variable names on the x and y axes. The color of the cells indicates the direction and strength of the correlation: red for positive and blue for negative. RMS, root mean square; HR, harmonic ratio; SampEn, sample entropy; RQA_rec, %recurrence in recurrence quantification analysis; RQA_det, %determinism in recurrence quantification analysis; sLE, short-term maximum Lyapunov exponent; AP, ML, VT, anterior-posterior, mediolateral, and vertical direction of the acceleration signal, respectively.


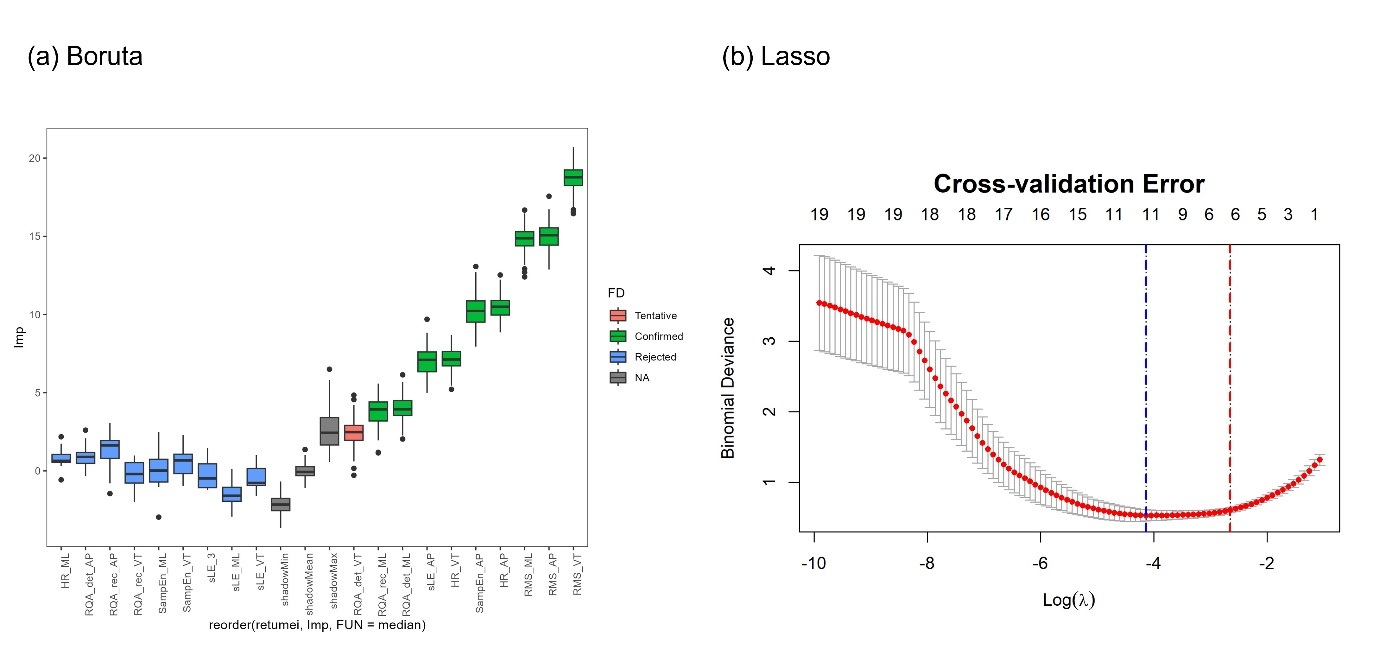


**Supplementary Fig. S2. Feature selection using Boruta and Lasso**

**(a) Feature importance estimated by the Boruta algorithm**
This plot shows the feature importance (Imp) evaluated using the Boruta algorithm. Green boxplots represent "Confirmed" features, blue represents "Rejected," red represents "Tentative," and gray indicates "NA" (not evaluated). Features are ordered based on the median importance.

**(b) Cross-validation results of Lasso regression**
This plot presents the cross-validation results of Lasso regression. The x-axis shows the regularization parameter log(λ), and the y-axis indicates binomial deviance. Red dots represent the cross-validation errors, and gray error bars represent the standard error. The blue dashed line indicates the minimum error criterion (lambda.min), and the red dashed line indicates the 1-standard-error rule (lambda.1se).


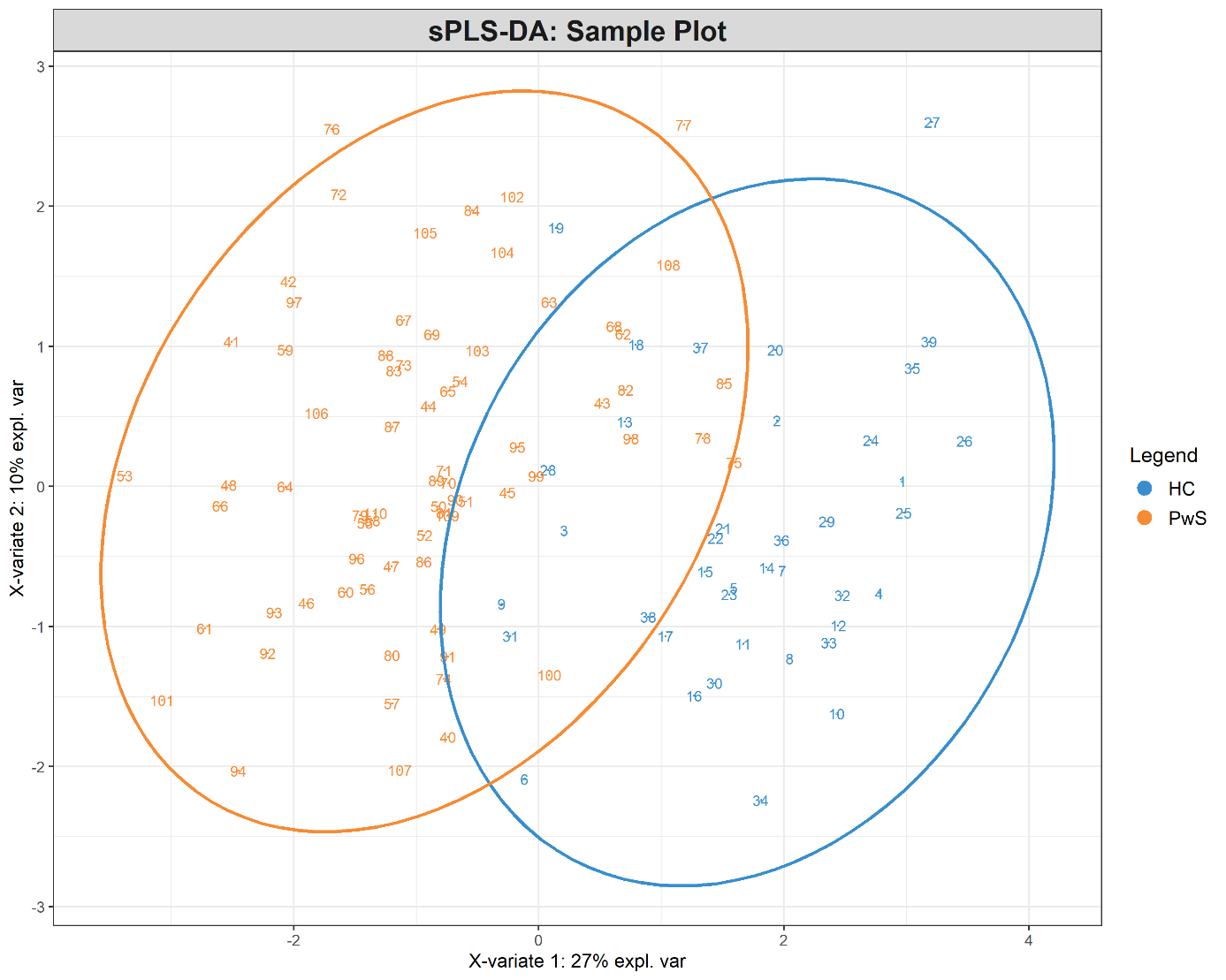


**Supplementary Fig.S3. Sample distribution in the sPLS-DA model (Component 1 vs. Component 2).**


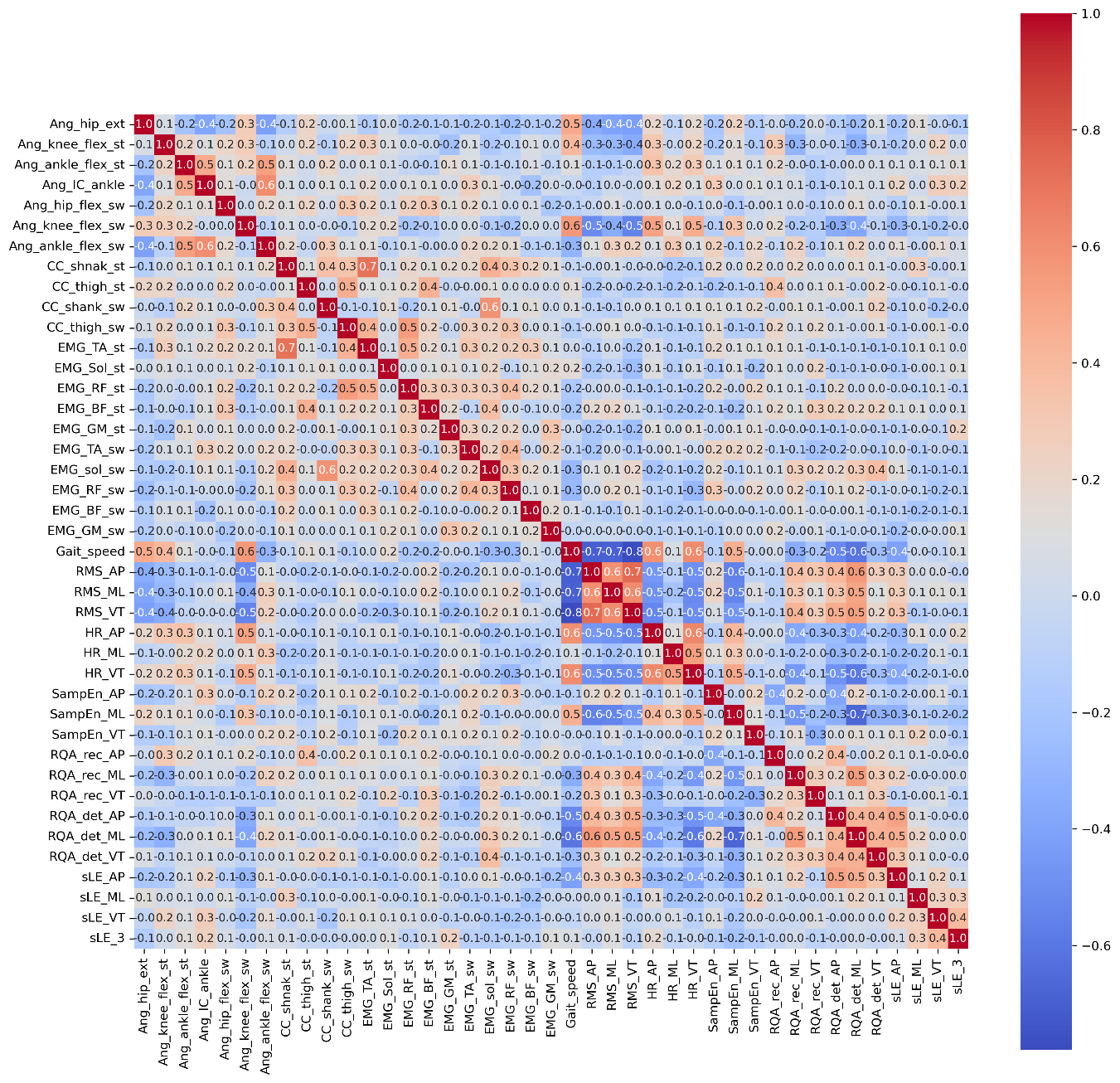


**Supplementary Fig. S4. Correlation heatmap (step2: even surface walking)**

The heatmap obtained using the Seaborn library's relplot function displays the correlation between initial features of the gait parameters during even surface walking. Each cell in the matrix represents the partial correlation between two variables, as indicated by the variable names on the x and y axes. The color of the cells indicates the direction and strength of the correlation: red for positive and blue for negative. CC, Co-contraction; EMG, Electromyography; TA, Tibialis Anterior; Sol, Soleus; RF, Rectus Femoris; BF, Biceps Femoris; GM, Gluteus Medius; RMS, root mean square; HR, harmonic ratio; SampEn, sample entropy; RQA_rec, %recurrence in recurrence quantification analysis; RQA_det, %determinism in recurrence quantification analysis; sLE, short-term maximum Lyapunov exponent; AP, ML, VT, anterior-posterior, mediolateral, and vertical direction of the acceleration signal, respectively.


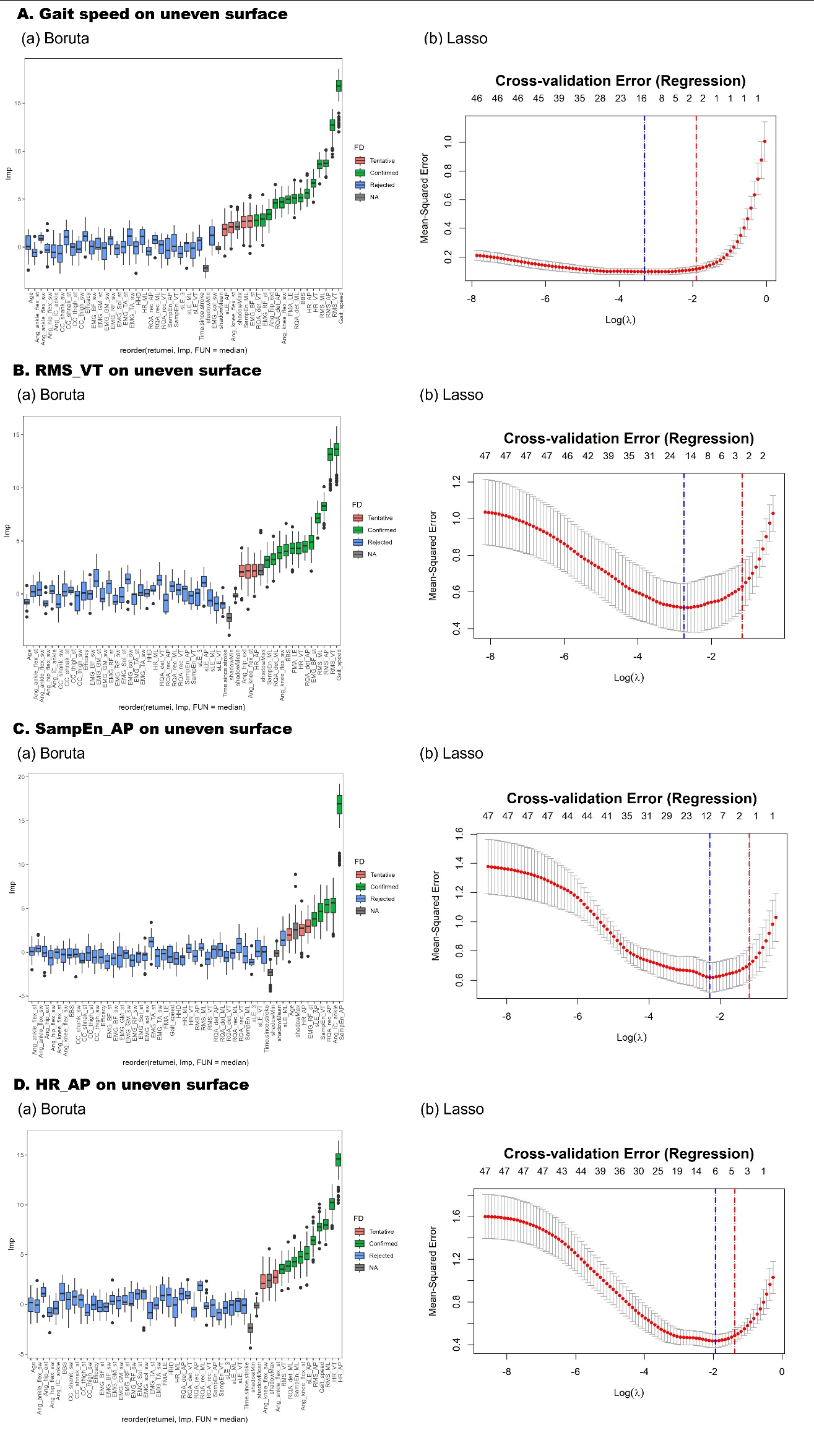


**Supplementary Fig. S5. Feature selection for each outcome on uneven surface walking using Boruta and Lasso algorithms**
A. Gait speed on uneven surface; B. RMS_VT on uneven surface; C. SampEn_AP on uneven surface; D. HR_AP on uneven surface.
RMS, root mean square; VT, vertical; SampEn, sample entropy; AP, anterior-posterior; HR, harmonic ratio.

**(a) Feature importance assessed by Boruta**
Feature importance (Imp) scores were evaluated using the Boruta algorithm. Green boxplots indicate "Confirmed" features, blue indicate "Rejected," red indicate "Tentative," and gray indicate "NA" (not assessed). Features are sorted by median importance.

**(b) Cross-validation results for Lasso regression**
Cross-validation results for Lasso regression. The x-axis represents the regularization parameter log(λ), and the y-axis represents binomial deviance. Red points indicate cross-validation errors; gray error bars represent standard error. The blue dashed line corresponds to the minimum error criterion (lambda.min), and the red dashed line corresponds to the one standard error criterion (lambda.1se).


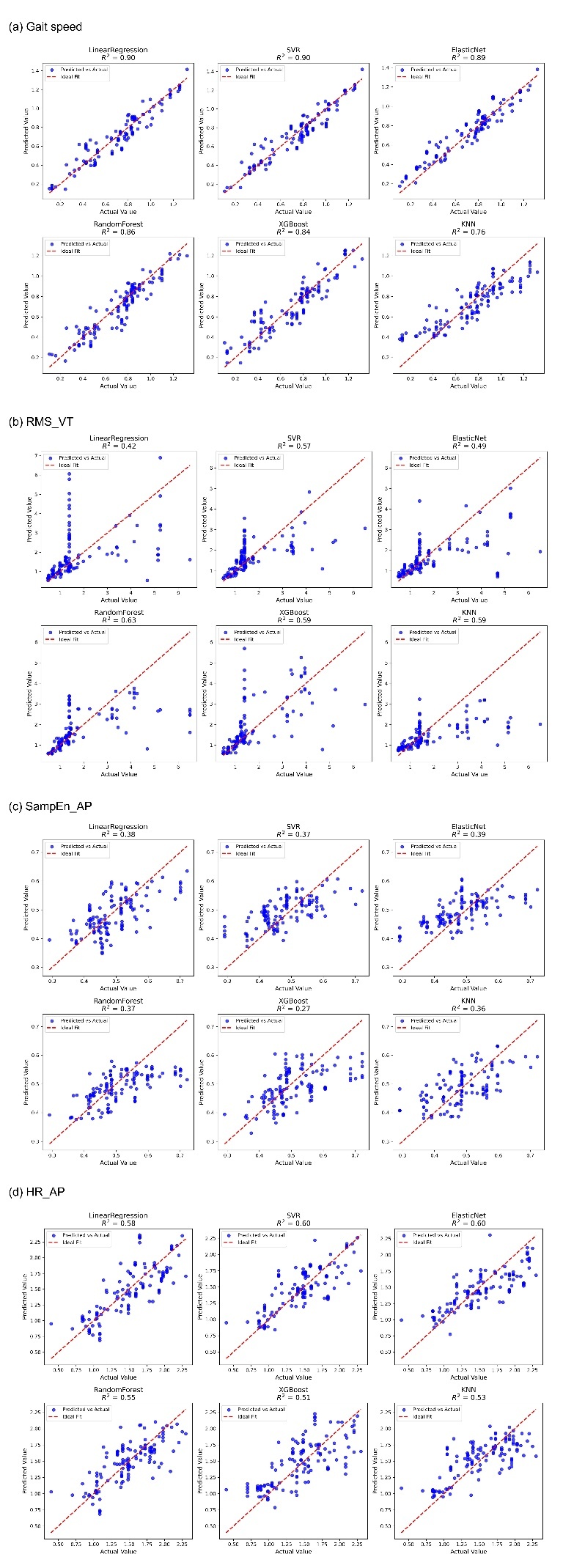


**Supplementary Fig. S6. Scatter plots of predicted versus actual values for each machine learning regression model** applied to the prediction of four uneven surface walking parameters:
(a) Gait speed, (b) RMS_VT, (c) SampEn_AP, and (d) HR_AP.
Each subplot shows the performance of six regression models: Linear Regression, Support Vector Regression (SVR), Elastic Net, Random Forest, XGBoost, and k-Nearest Neighbors (KNN). The red dashed line indicates the line of perfect prediction (*y = x*). The coefficient of determination is reported for each model. These plots correspond to the averaged model performance shown in Table 5 and complement the main manuscript by illustrating prediction accuracy across different algorithms.


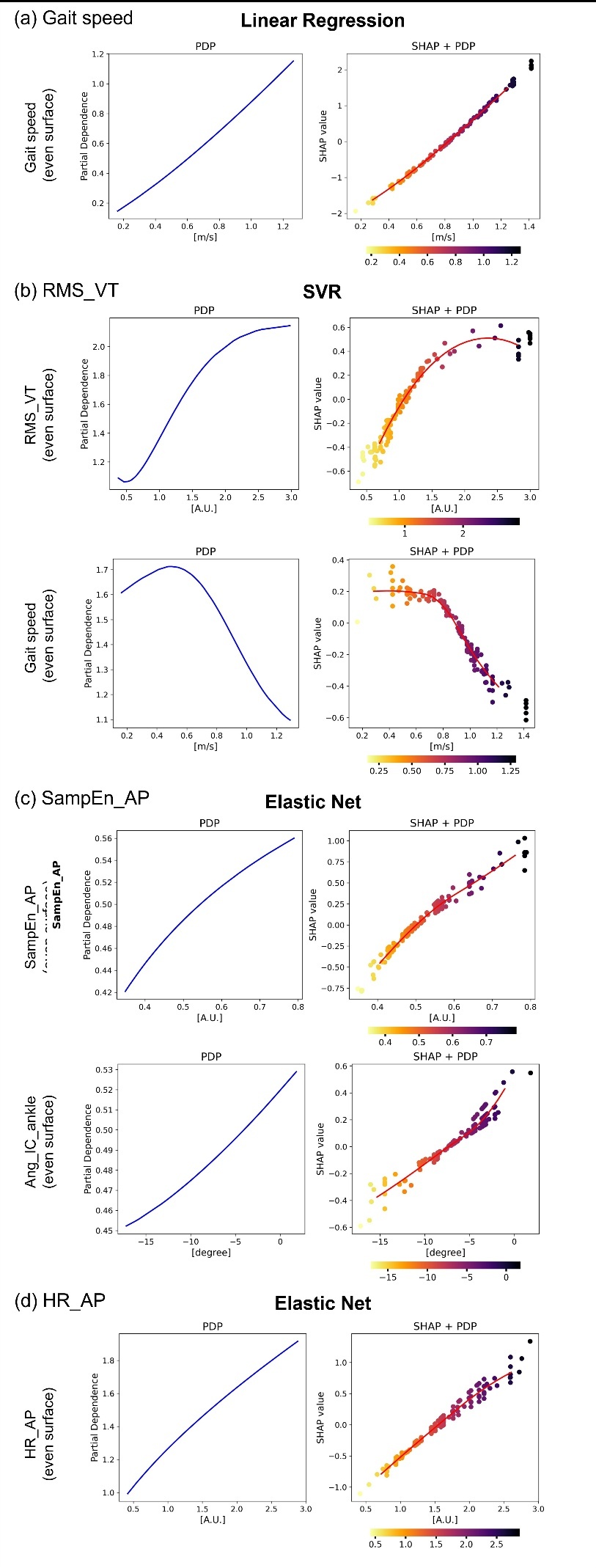


**Supplementary Fig. S7. Partial Dependence Plot (PDP) and SHAP Dependence Plot analyses based on linear models for predicting uneven-surface gait parameters.**
Each row corresponds to a target variable measured on uneven surfaces: (a) Gait speed (Linear Regression), (b) RMS_VT (SVR), (c) SampEn_AP (Elastic Net), and (d) HR_AP (Elastic Net). For each target, two even-surface gait predictors are shown: the same-name feature (top row) and an additional relevant feature identified via SHAP analysis (bottom row).
The left column displays PDPs showing the marginal effect of each feature on the prediction. The right column presents SHAP dependence plots, with individual data points colored by feature value. These results complement the main text figures based on nonlinear models and serve to illustrate the approximate linear relationships between key features and gait parameters.

RMS, root mean square; VT, vertical; SampEn, sample entropy; AP, anterior-posterior; HR, harmonic ratio.


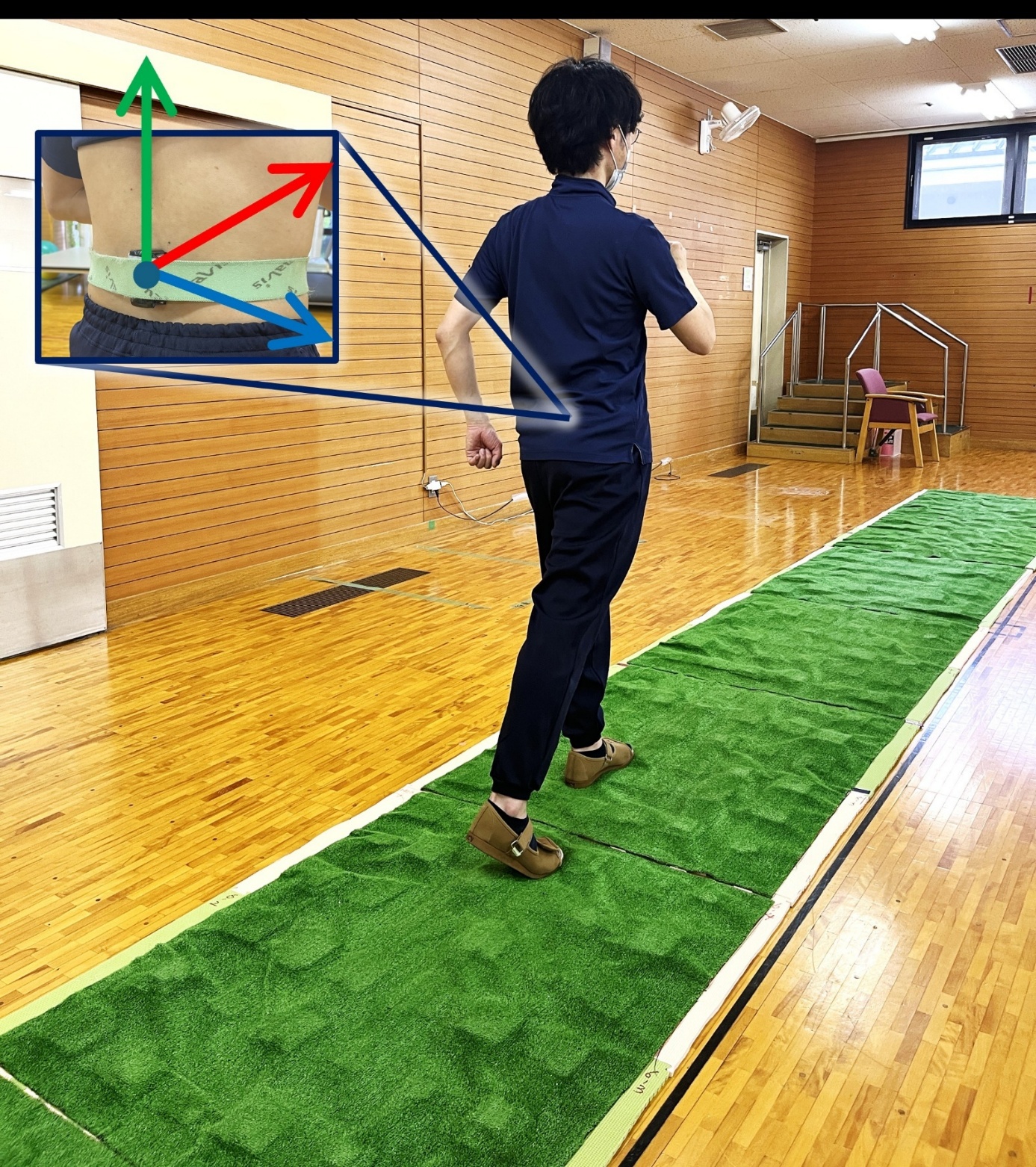


**Supplementary Fig. S8**. A participant walking on a custom-built uneven surface. An accelerometer is attached at the level of the third lumbar vertebra (L3) to assess gait stability.


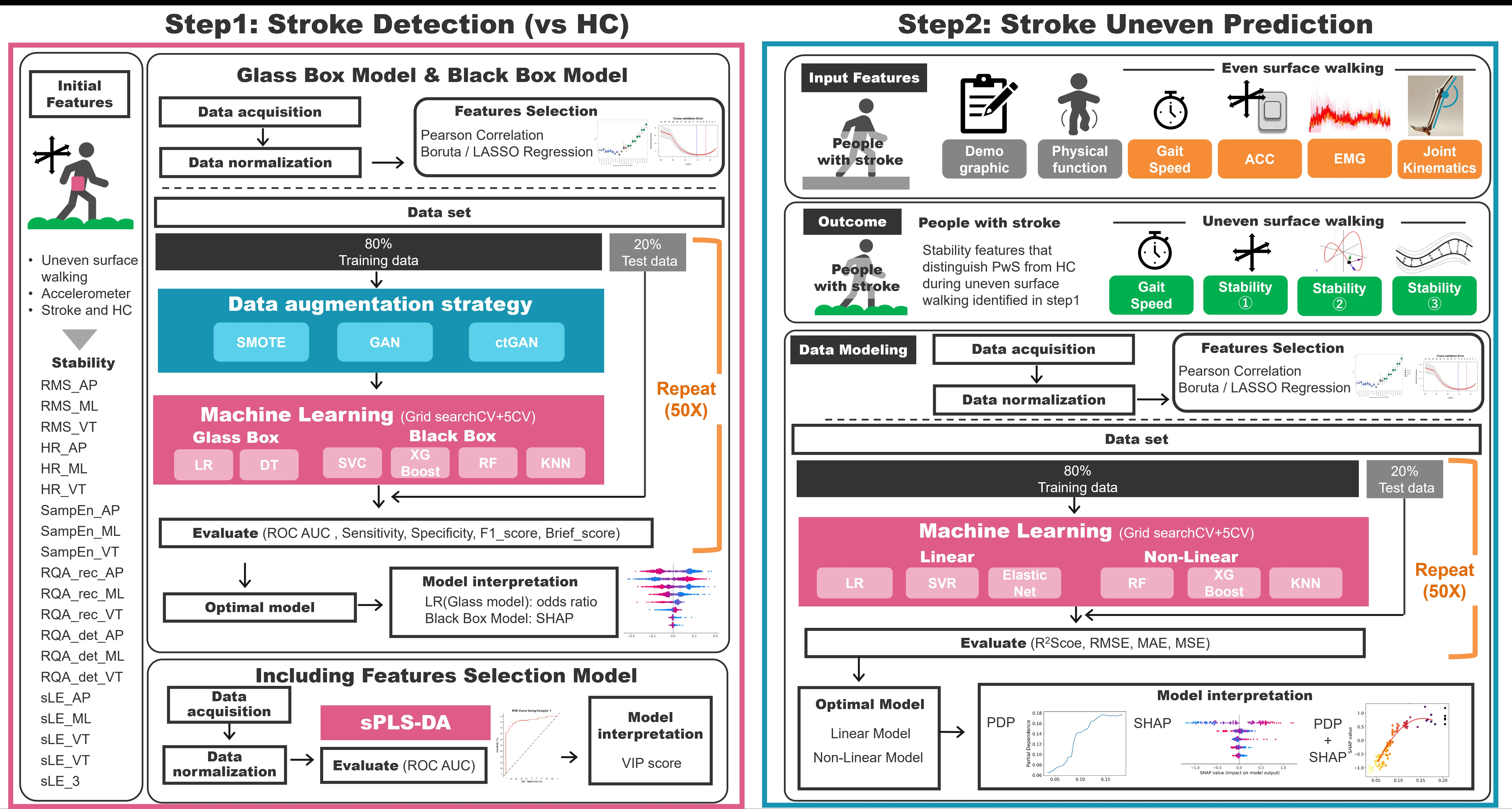


**Supplementary Fig. S9. Machine learning and data augmentation strategy.**HC, Healthy Control; RMS, root mean square; HR, harmonic ratio; SampEn, sample entropy; RQA_rec, percentage of recurrence in recurrence quantification analysis; RQA_det, percentage of determinism in recurrence quantification analysis; sLE, short-term maximum Lyapunov exponent; AP, ML, VT, anterior-posterior, mediolateral, and vertical directions of the acceleration signal, respectively; SMOTE, Synthetic Minority Over-sampling Technique; GAN, Generative Adversarial Network; ctGAN, Conditional Tabular Generative Adversarial Network; LR, Logistic Regression; DT, Decision Tree; SVC, Support Vector Classification; RF, Random Forest; KNN, k-nearest neighbors; SHAP, SHapley Additive exPlanations; ROC AUC, receiver operating characteristic–area under the curve; sPLS-DA, sparse partial least squares discriminant analysis; VIP score, variable importance in projection score; PDP, partial dependence plot.
